# The key role of meteorites in the formation of relevant prebiotic molecules in a formamide/water environment

**DOI:** 10.1038/srep38888

**Published:** 2016-12-13

**Authors:** Luca Rotelli, Josep M. Trigo-Rodríguez, Carles E. Moyano-Cambero, Eleonora Carota, Lorenzo Botta, Ernesto Di Mauro, Raffaele Saladino

**Affiliations:** 1 Biological and Ecological Department (DEB), University of Tuscia, 01100 Viterbo, Italy; 2 Institute of Space Sciences (CSIC-IEEC), Meteorites, Minor Bodies and Planetary Sciences Group, Campus UAB Bellaterra, Carrer de Can Magrans, s/n 08193 Cerdanyola del Vallés, Barcelona, Spain

## Abstract

We show that carbonaceous chondrite meteorites actively and selectively catalyze the formation of relevant prebiotic molecules from formamide in aqueous media. Specific catalytic behaviours are observed, depending on the origin and composition of the chondrites and on the type of water present in the system (activity: thermal > seawater > pure). We report the one-pot synthesis of all the natural nucleobases, of aminoacids and of eight carboxylic acids (forming, from pyruvic acid to citric acid, a continuous series encompassing a large part of the extant Krebs cycle). These data shape a general prebiotic scenario consisting of carbonaceous meteorites acting as catalysts and of a volcanic-like environment providing heat, thermal waters and formamide. This scenario also applies to the other solar system locations that experienced rich delivery of carbonaceous materials, and whose physical-chemical conditions could have allowed chemical evolution.

It is assumed that meteorites played a role in the origin of life, behaving as carriers of organic compounds during the Heavy Bombardment period on Earth^1^,^2^, before the earliest appearance of living organisms^3^. Carbonaceous chondrites are ancient space objects containing minerals condensed in the Solar Nebula or inherited from the delivery from nearby stars, partially modified by secondary processing histories^4^. They derive from asteroids that did not undergo planetary differentiation^5^ or from outer regions of the protoplanetary disk, and are characterized by reactive minerals, high water content and organic molecules, as amino acids, in the range of parts-per-billion (ppb)^6^,^7^. Theoretical and experimental models suggest that a part of the organic materials present in carbonaceous chondrites could have been synthesized before the parent body accretion and then evolve under hydrothermal alteration after accretion inside the parent body^8^ at temperature ranging from 100 °C to 150 °C due to radioactive phenomena^9^,^10^. It has been envisioned that water alteration in carbonaceous asteroids occurred under quite static conditions because they were “cooked” slowly and with restricted water availability (e.g. from bounded water in phyllosilicates, hydroxides or other minerals)^11^.

Current evidence suggests a significant and heterogeneous water content in the parent bodies of carbonaceous chondrites (0–25 wt. % H_2_O)^8^. Carbonaceous chondrites exhibit signs of static aqueous alteration that generated characteristic minerals in the fine-grained matrix, where reactions could take place as a function of the increasing complexity^12^.

Catalytic properties of meteorites in prebiotic chemistry were observed related to thermal processes of neat formamide (NH_2_CHO)^13^,^14^. Meteorites perform as catalysts better than terrestrial minerals, as exemplified by the one-pot formation of ribo- and 2’-deoxyribo nucleosides upon irradiation of NH_2_CHO with high-energy proton beams, mimicking the effect of solar wind^15^. Comments on the prebiotic relevance of NH_2_CHO are in Supplementary text SI # 1. Results on the use of HCN in presence of copper and UV light and involving a meteorite in the synthesis of some prebiotically relevant specific intermediates have been reported^16^.

The role of water in prebiotic chemistry is controversial, due to its nucleophile character inducing solvolysis reactions. NH_2_CHO and HCN are degraded in water, which might have limited their efficacy as prebiotic precursors^17^. In spite of water-caused instability and of possible dilution-related problems (potentially solved as in ref. 18), at one point molecular evolution shifted to water-based processes: extant genotypes and phenotypes are now profoundly adapted to and controlled by water.

Nucleosides are efficiently phosphorylated by reaction in NH_2_CHO or in water with phosphate minerals^19^ in conditions allowing spontaneous oligomerization, ligation and rudimentary ribozyme activities^20^ and references therein. Is there a positive role for water in these initial prebiotic synthetic processes? The chemical rationale behind this question relies on the hypothesis that, due to its complete solubility in water, NH_2_CHO might have provided, in the presence of carbonaceous chondrites, a smooth transition towards the aqueous chemistry onto which extant terrestrial life is based^21^.

## Results

We show that carbonaceous chondrites catalyze the synthesis of natural nucleobases, carboxylic acids, and amino acids from mixtures of NH_2_CHO and water at 140 °C. Two general scenarios were analyzed: thermal water (TW) and seawater (SW), both tested in the presence of NH_2_CHO and of one of six meteorites of the carbonaceous chondrite type: ALH 84028, EET 92042, MIL 05024, LAR 04318, GRO 95551, and GRO 95566. References, inorganic and organic composition, and cosmo-origin data of meteorites are detailed in Supplementary text SI # 2. NH_2_CHO was heated in the presence of meteorite materials and in the presence or in the absence of water, and the products were analyzed by GC-MS. Meteorites were treated before the use to remove possible organic contaminants. In two selected cases, ALH 84028 and EET 92042, they were used also as untreated samples. The water samples were carefully filtered before the use to remove any possible microbial and organic contamination. After the treatment, the water samples did not release detectable trace of organic substances. A similar result was also observed after heating ALH 84028 and water samples (distilled water DW, TW and SW) at 140 °C, further confirming that meteorite and water samples do not release detectable endogenous organics under the applied experimental conditions. The chromatographic profiles of reactions are in SI #6. The NH_2_COH/water ratio (40% water) was selected in accordance with results previously obtained in the thermal condensation of NH_2_COH with iron-copper sulfur minerals^22^, in order to compare the catalytic performance of meteorites relative to terrestrial minerals. Materials and methods are detailed in Methods and in SI # 3. The reaction of NH_2_CHO or NH_2_CHO/DW mixture in the absence of meteorite material afforded purine as the only recovered product, besides to traces of formic acid^23^ (Table 1, note a).

The samples of analyzed waters were: SW from the representative Mediterranean area of Montalto di Castro (Viterbo, Italy), and TW from the Bagnaccio volcanic spring area (Viterbo, Italy). The physical and chemical properties, and the ion composition of water samples are in SI # 4. The reaction performed with TW, SW and DW with NH_2_CHO in the absence of meteorites only afforded purine.

### ALH 84028

**Figure 1:**
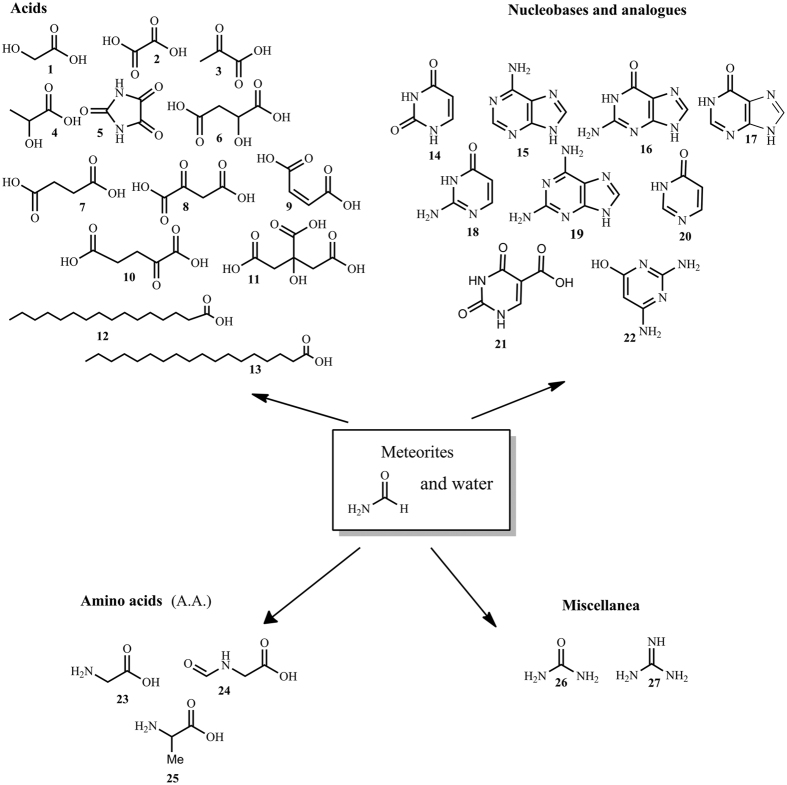
Prebiotic synthesis of biomolecules from meteorite and water in the presence of formamide. Products of thermal condensation from NH_2_CHO/water mixture in the presence of meteorites of carbonaceous chondrite sub-type. Experimental conditions: 1% meteorite, 59% NH_2_CHO, 40% water, 140 °C, 24 h.

In the presence of ALH84028 and DW, NH_2_CHO afforded a limited panel of compounds, including oxalic acid **2**, citric acid **11**, palmitic acid **12**, stearic acid **13**, glycine **23**, urea **26** and guanidine **27** (Fig. 1, Table 1, column D).

Better results were obtained in the presence of TW and SW, affording larger panels of products of biological relevance: carboxylic acids with increasing levels of structural complexity (from C2 to C16), as glycolic- **1**, oxalic- **2**, pyruvic- **3**, lactic- **4**, parabanic- **5**, malic- **6**, succinic- **7**, oxaloacetic- **8**, fumaric- **9**, ketoglutaric- **10**, citric- **11**, palmitic- **12**, and stearic- **13** acids; nucleobases and nucleobase analogues uracil **14**, adenine **15**, guanine **16**, hypoxanthine **17**, isocytosine **18** and 2,6-diamino purine **19**, purine and pyrimidine heterocycles 4 (3 H)-pyrimidinone **20**, uracil 5-carboxylic acid **21**, 2,4-diamino-6-hydroxypyrimidine **22**; amino acids glycine **23**, *N*-formyl glycine **24**, alanine **25**. Urea **26** and guanidine **27** were also detected (Fig. 1, Table 1, columns B and C).

Carboxylic acids are key intermediates in energy metabolism and for the synthesis of metabolites. Compounds **3**, **6**, **7**, **8**, **9**, **10** and **11**, are seven of the ten components of the Krebs cycle (KC), one of the most ancient cellular metabolic pathway. The relevance of the KC in the early production of energy and organics was postulated, invoking its prebiotic role in the fixation of carbon oxides from the atmosphere of the primitive Earth^24^. Compounds **1** and **2** are components of the anabolic alternative pathway of the KC in plants, bacteria, protists, and fungi (the glyoxilate cycle), **32** of the Cori cycle and gluconeogenesis. Long-chained carboxylic acids **12**–**13** are constituents of lipids. Carboxylic acids can be formed from NH_2_CHO by oligomerization to monoamino malonitrile (AMN) and diaminomalo nitrile (DAMN), followed by hydrolysis and successive redox processes^25^.

The complete set of RNA nucleobases was obtained (isocytosine **18** being bioisoster of cytosine), along with nucleobase analogues **17** and **19**–**21**, whose biological relevance was reviewed^26^. Reaction mechanisms for the formation of nucleobases from NH_2_CHO were reported^27^.

The presence of glycine **23**, *N*-formylglycine **24** and alanine **25**, is probably due to a NH_2_CHO-based Strecker-type synthesis (Strecker-cyanohydrin), in accordance with the recent theoretical observations on the key role of NH_2_CHO in the Miller-Urey synthesis of amino acids^28^. Glycine is classically the most abundant amino acid found in meteorites. *N*-formylglycine **24** is produced from **23** by a formylation process that mimics the peptidase activity involving “*in situ*” generated carbodiimide (not isolated in this case), urea **26** and guanidine **27**^29^. A schematic representation of the reaction pathways leading to nucleobases, carboxylic acids and amino acids from NH_2_CHO is in SI #8.

As a general trend, the reactions in TW or SW behaved as in the condensation of neat NH_2_CHO, suggesting that the presence of the meteorite ALH 84028 effectively increased the reactivity of the system, thus balancing the expected inhibition due to water dilution (Table 1, columns B and C versus column A). The treated or untreated ALH 84028 meteorite materials performed as catalyst very similarly in the reaction with neat NH_2_CHO (Table 1, column A). In some cases (oxalic acid, palmitic acid, adenine, glycine and alanine), the yield in the NH_2_CHO/water systems was higher than that obtained in neat NH_2_CHO (Table 1). The reaction in TW was always more efficient than in SW, affording a larger panel of products in higher yield (Table 1, columns B versus C).

### LAR 04318, EET 92042, GRO 95551, GRO 95566 and MIL 05024

The reaction was extended to other selected meteorites, LAR 04318, EET 92042, GRO 95551, GRO 95566 and MIL 05024, as reported in Tables 1 and 2. M/z values and peak abundances are in SI, Table C. Selected chromatograms and original m/z fragmentation spectra are in SI # 6 and SI # 7, respectively.

Even though activity relationships cannot be established due to the overall complexity of the systems, general considerations can be derived from the analysis of the reaction pattern in terms of quality and quantity of synthesized products. Irrespective of the experimental conditions, carboxylic acids were isolated in higher variety and yield in TW relative to SW, in agreement with data obtained with ALH 84028. In both TW and SW, the yield was comparable with that obtained using neat NH_2_CHO (Tables 1 and 2, columns B and C versus column A). Meteorites ALH 84028, LAR 04318 and GRO 95566 produced the highest number of derivatives (ten and eight carboxylic acids, respectively). Some of these derivatives were obtained with high selectivity, as malic acid (observed only with LAR 04318) and oxaloacetic acid (observed only with EET 92042 and GRO 95566). In terms of isolated compounds, LAR 04318 and GRO 95566 afforded the highest total amount of carboxylic acids (c.a. 210 μg/mL and 98 μg/mL, respectively), followed by ALH 84028, EET 92042 and MIL 05024, which showed a similar behavior (c.a. 70 μg/mL). A high quantitative efficacy in the synthesis of carboxylic acids did not necessarily coincide with a high variety of synthesized products (i. e., Table 1, ALH 84028 versus LAR 04318).

The yield of nucleobases was lower than that of carboxylic acids. In agreement with the general trend observed for carboxylic acids, nucleobases and nucleobase analogues were synthesized in higher yield in TW than in SW (Tables 1 and 2). ALH 84028 and GRO 9556 were the only meteorites that yielded the complete set of RNA nucleobases. Adenine and guanine were the most represented nucleobases, isocytosine being obtained only with ALH 84028 and GRO 95566. In terms of isolated compounds, ALH 84028, GRO 95566 and EET 92042 afforded the highest total amount of nucleobases and nucleobase analogues in NH_2_CHO/TW (c.a. 9.5 μg/mL, respectively), followed by GRO 95566 (c.a. 5.5 μg/mL). Occasionally, nucleobases were synthesized in yield higher in NH_2_CHO/TW than in neat NH_2_CHO, as observed for guanine and adenine in the presence of GRO 95566 and ALH 84028, respectively. Glycine, *N*-formyl glycine and alanine were always synthesized, EET 92042 being the best catalyst, followed by ALH 84028 and MIL 05024. Again, treated or untreated EET 92042 materials performed as catalyst very similarly in the reaction of neat NH_2_CHO.

## Conclusions

A careful comparison of the relationships between the products obtained and the meteorite used as catalyst shows a complex scenario. For example, the EET 92042 (CR2) meteorite catalyzes the synthesis of a panel of products whose qualitative composition (namely, the panel of products and their relative abundance) is similar to the panel of its endogenous organics. At the contrary, ALH 84028 (CV3) catalyzes the formation of a panel of products richer than that observed in the original sample. Thus, the catalytic role played by meteorites in the transformation of NH_2_CHO is tuned by the specific reaction environment, depending on the history and composition of the catalyst.

These data hint to the important role that chondrites could have played in an early Earth subjected to a strong projectile flux. They also indicate that, even after ablation of the volatile organic phases from the impactants during their fall, the mineral components that eventually reached the ground could have promoted the catalysis of organics in an environment that was more hydrous and oxidizing than the one they had in their progenitor asteroids^30^.

TW and, to lower extent, SW were more reactive than DW. The types of water we used largely differ for their inorganic composition (as detailed in SI, Tables A and B) and for their pH (pH 6.0 for TW versus pH 8.1 for SW). TW shows concentrations of alkaline earth metals Ca, Mg, Sr and Ba, largely higher than SW. In addition, transition metal ions Mn and Fe were detected only in TW. Transition metal ions very efficiently coordinate aliphatic amides, achieving their maximum coordination number^31^. Alkaline earth metals and transition metal ions coordinate the nucleophilic centers in NH_2_CHO, to yield complexes with different geometries and stabilities, which are known to lose water in the presence of high energy sources to yield HCN^32^, one of the key intermediate in the synthesis of both nucleobases and carboxylic acids (SI #8). Different transition metal compounds have been recently reported as efficient catalysts for the prebiotic synthesis of a large panel of prebiotic molecules from NH_2_CHO in geochemical scenarios^33^. Hydrothermal systems are accepted models of prebiotic environments, and several organics have been detected in abiotic hot springs^34^.

The selectivity of the reactions was tuned by the nature of each chondrite, probably as a function of the differently available amounts of minerals acting as catalysts. Glycine, *N*-formyl glycine and alanine were isolated in the highest yield in the presence of EET 92042 (classified as CR2 chondrite), followed by ALH 84028 (CV3) and MIL 05024 (CO3).

This order of reactivity is in agreement with the observed and theoretically calculated ability of carbonaceous chondrites to perform the Strecker-type synthesis, that is CR> CV3 (CK3)> CO3^35^. This observation prompts us to clarify our interpretation of the data obtained, as follows: the syntheses of prebiotically relevant compounds was observed in a system composed of a reactive substrate (NH_2_CHO), of catalysts (chondrite materials) in a typically Earth-like environment (heat + waters, thermal or otherwise). Thus, we frame our synthetic system in an earliest planet Earth scenario. Nevertheless, the very fact that the syntheses of certain compounds bona fide occurred according to mechanisms that were both theorized to be possible and observed to have occurred in meteorites during their non terrestrial history (i.e., Strecker-type synthesis of aminoacids, ref. 4), points that these processes are not exclusively terrestrial.

The CR chondrite group affords the highest yield of amino acids, greater by several orders of magnitude than any other subclass. This subclass is generally associated with alteration temperatures in the broad range of 0 °C to 240 °C^36^. In view of recent results, the catalytic role exhibited by the CR mineral components is not surprising as it is an extraordinarily pristine group, being identified as source of primitive organics^37^,^38^.

Deviations from this reaction pattern were observed for carboxylic acids and nucleobases. LAR 04318 (CK4), ALH 84028 (CV3), EET 92042 (CR2) and GRO 95566 (C2-ung) were found to be the most active catalysts for these compounds. The difference between the mechanism of a Strecker-type synthesis relative to that of the formation of carboxylic acids and nucleobases from NH_2_CHO may account for this result (SI #8).

The complexity of the relationships between organics, water and early stages of aqueous alteration in pristine chondrites was described^8^. This study confirms the view that reactive minerals could have acted as catalysts promoting increasing organic complexity in chemical evolution. In particular, some secondary minerals were found to be the product of primordial aqueous alteration, during a short first stage of water release due to radiogenic heating^39^, and often the minerals formed exhibit clear features of static aqueous alteration with limited water availability^12^.

NH_2_CHO/water solutions were recently shown to form a multipurpose reactive mixture in a totally different reaction system^33^. Unlike recent theoretical argumentation of the contrary^40^, the data described here and in ref. 33 show that NH_2_CHO/water is a flexible and fertile prebiotic incubator.

These study highlights the role of water, of thermal processes and of one-carbon atom precursors (such as NH_2_CHO) in the endogenous internal prebiotic chemistry of meteorites leading to the diverse organic compounds detected in chondrites, and opens a door to the understanding of the appearance of life in other planetary bodies that experienced an efficient delivery of carbonaceous chondrite materials, as Mars, Titan or Europa.

## Methods

Approximately 50 mg of the meteorite stone obtained after removal of the fusion crust were ground in an agate mortar and treated with NaOH 0.1 N (1.0 mL) and CHCl_3_-MeOH mixture (3.0 mL; 2:1 v/v), followed by sulphuric acid 0.1 N (1.0 mL) and CHCl_3_-MeOH mixture (3.0 mL; 2:1 v/v). ALH 84028 (CV3) and EET 92042 (CR2) were also used as untreated samples. The reactions were performed by heating freshly distilled NH_2_COH (1.0 mL) at 140 °C for 24 hours in the presence of the appropriate meteorite sample (1.0% by weight relative to NH_2_COH) and 40% in weight of distilled water DW, thermal water TW or sea water SW. The water samples were carefully treated before the use to remove any possible microbial and organic contamination by filtration on 0.20 μm *Minisart Sartorius* (catalogue number 16534; Sterile-E0), followed by extractions with EtOAc (20 ml; ×3). Reactions with neat NH_2_COH, with NH_2_COH and water without the meteorite sample, or with meteorite (ALH 84028) and water (DW, TW and SW) without NH_2_COH were also performed as references. At the end of the reaction the meteorite was recovered by centrifugation (6000 rpm, 10 min, Haereus Biofuge) and washed with MeOH. The excess NH_2_COH and MeOH were then removed by distillation (40 °C, 4 × 10^−4^ barr). The crude product was analyzed by gas-chromatography associated to mass-spectrometry (GC-MS) after treatment with *N,N*-bis-trimethylsilyl trifluoroacetamide in pyridine (620 μL) at 60 °C for 4 h in the presence of betulinic acid acid [3β-hydroxy-20^29^-lupaene-oic acid] as internal standard (0.3 mg). Materials and methods are detailed in SI # 3.

## Additional Information

**How to cite this article:** Rotelli, L. *et al*. The key role of meteorites in the formation of relevant prebiotic molecules in a formamide/water environment. *Sci. Rep.* **6**, 38888; doi: 10.1038/srep38888 (2016).

**Publisher's note:** Springer Nature remains neutral with regard to jurisdictional claims in published maps and institutional affiliations.

## Supplementary Material

Supplementary Information

## Figures and Tables

**Table 1:** Products of thermal condensation from NH_2_CHO/water mixtures in the presence of ALH 84028, LAR 04318 and EET 92042 meteoritic components.

			ALH 84028 (CV3)				LAR 04318 (CK4)			EET 92042 (CR2)		
Product^a^			A	B	C	D	A	B	C	A	B	C
**Acids (μg)**	C2	Glycolic ac. **1**	25,0 (25,1)^b^	10,3	9,8	—	—	—	—	9,8 (9,8)^b^	—	—
		Oxalic ac. **2**	traces	5,2	9,0	1,3	125,0	95,0	60,3	—	—	—
	C3	Pyruvic ac. **3**	2,5 (2,5)	1,8	—	—	25,0	25,1	—	68,4 (68,3)	66,8	46,3
		Lactic ac. **4**	28,7 (28,6)	12,5	11.3	—	—	—	—	—	—	—
		Parabanic ac. **5**	—	—	—	—	16,7	8.9	—	—	—	—
	C4	Malic ac. **6**	—	—	—	—	6,0	4,4	—	—	—	—
		Succinic ac. **7**	6,6 (6,6)	0,1	traces	—	4.9	2,3	2,5	—	—	—
		Oxaloacetic ac. **8**	—	—	—	—	—	—	—	3,0 (3,0)	2.5	6,5
		Fumaric ac. **9**	0,07 (0,06)	0,05	traces	—	—	—	—	—	—	—
	C5	Ketoglutaric ac. **10**	0,08 (0,08)	0,07	traces	—	—	—	—	—	—	—
	C6	Citric ac. **11**	4,8 (4,8)	3,3	1,3	6,9	16,4	6,0	3,2	5.6 (5,7)	3,4	2,1
	C16	Palmitic ac. **12**	20,9 (20,8)	25,2	21,2	7,4	47,4	25,9	8,1	—	—	—
		Stearic ac. **13**	37,7 (37,7)	14,5	9,0	7,9	83,1	42,6	33,1	—	—	—
**Heterocycles (μg)**	Uracil **14**		9,7 (9,7)	3,6	2,5	—	14,4	0,1	—	—	—	—
	Adenine **15**		1,5 (1,5)	2,4	1.3	—	—	0,5	—	3,3 (3,3)	1,4	1,1
	Guanine **16**		1,4 (1,5)	1,2	1,2	—	—	—	—	3,1 (3,1)	2,9	2.9
	Hypoxanthine **17**		3,7 (3,8)	1,3	—	—	—	—	—	5.1 (5,1)	5,3	2,7
	Isocytosine **18**		12,6 (12,5)	0,9	0,1	—	—	—	—	—	—	—
	2,6-Diaminopurine **19**		—	—	—	—	—	—	—	7,5 (7,5)	6,3	5,1
	4 (3 H)-pyrimidinone **20**		—	—	—	—	—	—	—	4,8 (4,7)	1,7	1,6
	Uracil 5-carboxylic ac. **21**		0,4 (0,4)	0,4	—	—	—	—	—		—	—
	2,4-diamino-6-hydroxypyrimidine **22**		25,9 (25,9)	0.6	—	—	35,5	—	—	8,1 (8,1)	7,33	6,1
**A.A. (μg)**	Glycine **23**		14,3 (14,4)	25,3	23,2	1,1	0,9	1,4	1,1	10,0 (9,9)	28,3	25,1
	Formyl glycine **24**		48,7 (48,6)	3,4	3,3	—	traces	56,7	29,8	51 (51)	12,5	8,1
	Alanine **25**		12,1 (12,1)	6,3	6,0	—	traces	2,2	1,4	7,1 (7,2)	9,8	9,0
**Mix (μg)**	Urea **26**		0,9 (0,9)	0,5	—	1,0	1,1	0,5	—	—	—	—
	Guanidine **27**		50,1 (50,1)	33,4	28,7	6,5	64,6	37,4	35,6	58,9 (58,9)	15,4	10,3

^a^The reaction of NH_2_CHO in the absence of meteorite material afforded purine (3.4 mg) as the only recovered product. Similarly, the reaction of NH_2_CHO/DW mixture in the absence of meteorite material afforded purine (0.2 mg) besides to traces of formic acid. ^b^Reaction performed with untreated meteorite material. A: NH_2_CHO and meteorites without water. B: NH_2_CHO and meteorites in the presence of thermal water. C: NH_2_CHO and meteorites in the presence of seawater. D: NH_2_CHO and meteorites in the presence of distilled water. A. A. amino acids. The data are the mean values of three experiments with standard deviation less than 0.1%. Products are given in μg per mL of NH_2_CHO.

**Table 2:** Products of thermal condensation from NH_2_CHO/water mixtures in the presence of GRO 95551, GRO 95566 and MIL 05024 meteorites.

			GRO 95551 (C-ung)			GRO 95566 (C2-ung)			MIL 05024 (CO3)		
Product			A	B	C	A	B	C	A	B	C
**Acids (μg)**	C2	Glycolic **1**	—	—	—	—	—	—	0,5	0,5	—
		Oxalic **2**	6,2	5,8	5,0	2,5	—	—	—	—	—
	C3	Pyruvic **3**	8,3	10,9	5,9	46,9	10,9	11,0	16,7	13,6	12,4
		Lactic **4**	6.5	2.9	2,3	6,8	9,4	2,2			
	C4	Succinic **7**	3,0	2,5	1,4	2.9	—	—	12.9	11.5	8.3
		Oxaloacetic **8**	—	—	—	1,4	1.5	—	—	—	—
		Fumaric **9**	13.3	10.2	9.8	—	—	—	—	—	—
	C5	Ketoglutaric **10**	6,8	1,2	—	—	—	—	—	—	—
		Citric **11**	—	—	—	4,3	3,8	0,3	—	—	—
	C16	Palmitic **12**	25,3	10,2	10,0	35,9	39,2	32,0	19,4	18.3	13.6
		Stearic **13**	19,7	15,6	12,3	24,6	33,6	29,1	37,7	26,3	31,7
	Uracil **14**		—	—	—	6,0	0,2	0,2	—	—	—
**Heterocycles (μg)**	Adenine **15**		3,1	0,7	0,8	1,3	0,4	0,1	—	—	—
	Guanine **16**		1.4	1.2	1,2	1,7	3,5	1,1	5,0	2,3	1,8
	Hypoxanthine **17**		4,9	3.6	2,2	4,6	3,7	2.9	2,2	1,7	1,1
	Isocytosine **18**		—	—	—	3,3	2,1	1,8	—	—	—
	2,6-Diaminopurine **19**		7,1	3,6	2,1	8,3	—	—	—	—	—
	4 (3 H)-pyrimidinone **20**		1,3	9,5	—	3,8	4,2	2,4	4.3	3,4	2,1
	Uracil 5-carboxylic ac. **21**		5,2	—	—	13,6	—	—	—	—	—
	2,4-diamino-6-hydroxypyrimidine **22**		32,5	2,7	2,8	12,4	8,9	6,6	—	—	—
**A. A. (μg)**	Glycine **23**		3.3	2.2	2.0	5.9	3,3	2,2	3,7	3,1	2.8
	Formyl glycine **24**		6.5	6.0	5.3	1,1	4,2	3.1	6,6	6.9	5,7
	Alanine **25**		4,1	3,7	3,5	6,2	3,2	1,8	3,4	3,2	3,0
**Mix (μg)**	Urea **26**		—	—	—	2,0	—	—	0,6	0,3	0,3
	Guanidine **27**		85,3	55,9	24,7	2,8	—	—	6,6	5,9	—

A: NH_2_CHO and meteorites without water. B: NH_2_CHO and meteorites in the presence of thermal water. C: NH_2_CHO and meteorites in the presence of sea water. A. A. Amino acids. The data are the mean values of three experiments with standard deviation less than 0.1%. Products are given in μg (per mL of NH_2_CHO).
