# Supplementary material for: The key role of meteorites in the formation of relevant prebiotic molecules in a formamide/water environment
Source: Sci Rep. 2016 Dec 13;6:38888. doi: 10.1038/srep38888 (PMC5153646; doi:10.1038/srep38888)
Supplement: Supplementary Information [file srep38888-s1.doc]

**The key role of meteorites in the formation of relevant prebiotic molecules in a formamide/water environment**

Luca Rotelli**,**^1^ Josep M. Trigo-Rodríguez,^2^ Carles E. Moyano-Cambero,^2^ Eleonora Carota,^1^ Lorenzo Botta,^1^ Ernesto Di Mauro,^1,*^ Raffaele Saladino^1^,*

*^1^Biological and Ecological Department (DEB), University of Tuscia, 01100 Viterbo, Italy. ^2^Institute of Space Sciences (CSIC-IEEC), Meteorites, Minor Bodies and Planetary Sciences Group, Campus UAB Bellaterra, Carrer de Can Magrans, s/n 08193 Cerdanyola del Vallés, Barcelona, Spain.*

Corresponding authors. Email: [saladino@unitus.it](mailto:saladino@unitus.it), ernesto.dimauro@uniroma1.it

**Supplementary Materials-Supplementary text**

**SI # 1. Prebiotic relevance of NH_2_COH**

Formamide NH_2_COH (the simplest one-carbon amide in nature) is formally the condensation product of HCN and H_2_O, which are two of the most ancient compounds deemed to be among the first molecules formed on our planet (41). NH_2_COH is largely diffused in the universe, having been detected in Kparsec-wide interstellar clouds (42), and in several space objects (43). Space and terrestrial syntheses of NH_2_CHO under a variety of conditions have been previously described and explained (44). For instance, NH_2_COH has been produced in the past by reaction of ammonia and formic acid. While ammonia is generally accepted to be a major component of the primeval atmosphere (45), formic acid is the most abundant product formed in the classical Miller–Urey experiment (46). At difference from HCN, NH_2_CHO is liquid between 4 and 210°C, making it particularly suited to temperature-induced concentration phenomena (47). Alternatively it could be concentrated by formation of eutectic-phase in ice (48). Conditions for its extreme concentration have been recently reported in hydrothermal pores to form prebiotic nucleobases (49). The synthesis of complex organic compounds of biological relevance from NH_2_COH under plausible prebiotic conditions has been described and reviewed (50).

**SI # 2. References, composition, and cosmo-origin data of carbonaceous chondrites**.

We have analyzed the catalytic effect of six meteorites of the carbonaceous chondrite type, namely Allan Hills 84028 (ALH 84028, class CV3), Elephant Moraine 92042 (EET 92042, class CR2), Miller Range 05024 (MIL 05024, class CO3), Larkman Nunatak 04318 (LAR 04318, CK4), Grosvenor Mountains 95551 (GRO 95551, C-ung), and Grosvenor Mountains 95566 (GRO 95566, class C2-ung). They are characterized by the group designation CV, CR, CO, CK, C-ung e C2-ung, followed by a number indicating the petrologic type concerning the degree of internal parent body thermal and aqueous processing. Petrologic type 1 chondrites are the most aqueously altered. Petrologic type 2 meteorites have undergone significant degrees of aqueous alteration. Petrologic type 3 meteorites most closely resemble the solar nebulae in which they formed; they have undergone relatively little thermal metamorphism, no hydration. Petrologic types 4–6 indicate increasing degrees of thermal metamorphism (in other words: heating).

About the subclass designation of meteorites used in this study, the CV and CO meteorites are all petrologic type 3. These meteorites are associated with peak metamorphic temperatures ranging from 200–600°C, though CV3 peak temperatures at a much lower 50°C have been also reported (51). These chondrites are thought to be the most pristine samples of material available from the time of the solar system formation. They are relatively unaltered, neither via aqueous alteration nor via thermal metamorphism (which occurs at higher temperatures, normally at c.a. 500°−950°C in the absence of water). Lastly, the CK meteorites encopass petrologic types 3–6 and are associated with temperatures from 250°C–600°C (52). Usually, CK4–6 meteorites were found to have very low abundances of amino acids. GRO 95551 C-ung is a meteorite whose characteristics, given the high percentage of enstatite, are intermediate between the carbonaceous chondrites and the ordinary. The condrules of this meteorite have the following composition: from 41.1 to 62.5% of silicon dioxide, from 20.1 to 35.7% of aluminum oxide, from 3.8 to 19.0% of calcium oxide and traces of sodium oxide, their matrix being rich in nickel and iron troilite (53). Finally, GRO95566 is a carbonaceous meteorite that cannot be classified in one of the eight canonical groups and for this reason, is inserted in the C2-ung type (54). It contains numerous small chondrules (up to 0.6 mm in diameter), with traces of nickel-iron and is characterized by abundant hydrated minerals. Its matrix consists primarily of serpentine [(Mg, Fe)_3_Si_2_O_5_(OH)_4_, predominantly ferrous] (55).

From the analysis of a limited set of meteorites (CI, CM and C3), the total carbon content in carbonaceous chondrites was estimated in the range from 0.22 % to 3% in weight. At least 70% of the total carbon content is the insoluble fraction (Kerogen-like material), which can be detected only after dissolution of the inorganic matrix with HF-HCl mixture. The resting 30% corresponds to the soluble fraction containing different families of organic compounds. As a general trend, carboxylic acids are the most abundant compounds, amino acids and nucleobases being recovered generally in an order of magnitude smaller (56). In our experimental conditions, 1 mg of meteorite (previously treated to remove soluble endogenous organics) was used as catalyst. The expected amount of endogenous organics in this sample is expected to be in the order of ng (or even lower) per single substance. The abundance of soluble organic compounds in meteorite of the CR2 type (e.g. EET 92042) has been reported in detail (57). In this latter case, carboxylic acids were detected in the range of 1.0 μmol/g of starting material, while aminoacids were recovered in the range of 2.0 nmol/g (58, 59). The abundance of soluble organic compounds in meteorite of the CV type is reported (60), being significantly lower than that of meteorites of the CM type. The carbon and nitrogen content in meteorites of the CK4 type has been reported in ref. 61. Data for the composition of meteorites of the CO3 type are in ref. 62.

**SI # 3. Materials and Methods**

The carbonaceous chondrites analysed here were requested to the Johnson Space Center facility in the framework of two Spanish research projects to identify pristine meteorites in the NASA Antarctic collection. Approximately 50 mg of the stone obtained after removal of the fusion crust were ground in an agate mortar. The extraction of the meteorite powder to remove endogenous organics was carried out in two steps as previously reported (63). Briefly, the sample was treated with NaOH 0.1 N (1.0 mL) and CHCl_3_-MeOH mixture (3.0 mL; 2:1 v/v), followed by sulphuric acid 0.1 N (1.0 mL) and CHCl_3_-MeOH mixture (3.0 mL; 2:1 v/v). Between steps the powder was precipitated by a brief low-speed centrifugation (6000 rpm, 10 min., Haereus Biofuge) and the supernatant phase was decanted. After the treatment, the material did not release any trace of organic substances. Untreated meteorites ALH 84028 (CV3) and EET 92042 (CR2) were also used as selected samples in the experiments with neat NH_2_COH to evaluate the possible effect of the treatment on the catalytic performance of the mineral. As reported in Table 2 (main text), treated and untreated meteorites performed very similarly, in accordance with previous studies conducted with different type of meteorites (63). The reactions were performed by heating freshly distilled NH_2_COH (1.0 mL) at 140°C for 24 hours in the presence of the appropriate meteorite sample (1.0% by weight relative to NH_2_COH) and 40% in weight of distilled water DW, thermal water TW or sea water SW. The NH_2_COH/water ratio was selected in accordance with results previously obtained in the thermal condensation of NH_2_COH with iron sulfur and iron copper sulfur minerals (22). In this latter case, no prebiotic syntheses were observed at more than 30% water. On the basis of these data, the reaction were performed in 40% water in order to stress the catalytic performance of meteorites relative to terrestrial minerals. The water samples were carefully treated before the use to remove any possible microbial and organic contamination. In particular, TW and SW (100 mL) were nano- and micro-filtered on 0.20 μm *Minisart Sartorius* (catalogue number 16534; Sterile-E0) , followed by extractions with EtOAc (20 ml; x 3). After the treatment, the water samples did not release any trace of organic substances. Reactions with neat NH_2_COH, with NH_2_COH and water without the meteorite sample, or with meteorite (ALH 84028) and water (DW, TW and SW) without NH_2_COH were also performed as references. At the end of the reaction the meteorite was recovered by centrifugation (6000 rpm, 10 min, Haereus Biofuge) and washed with MeOH. The excess NH_2_COH and MeOH were then removed by distillation (40°C, 4×10^−4^ barr). The crude product was analyzed by gas-chromatography associated to mass-spectrometry (GC-MS) after treatment with *N*,*N*-bis-trimethylsilyl trifluoroacetamide in pyridine (620 μL) at 60°C for 4 h in the presence of betulinic acid acid [3β-hydroxy-20(29)-lupaene-oic acid] as internal standard (0.3 mg). Mass spectrometry was performed by the following program: injection temperature 280°C, detector temperature 280°C, gradient 100°C×2min, 10°C/min for 60 min. To identify the structure of the products, two strategies were followed. First, the spectra were compared with commercially available electron mass spectrum libraries such as NIST (Fison, Manchester, UK). Secondly, GC-MS analysis was repeated with standard compounds. All products have been recognized with a similarity index (S.I.) greater than 98% compared to the reference standards. The abundance of peaks is reported in parentheses in the relevant Table C in SI # 5.

**SI # 4. Composition and historical data of SW and TW samples**.

The chemical constituents were identified by liquid ion chromatography (IC 761 Metrom) and determinations of minor and trace elements were carried-out by quadrupole ICP-MS analysis (Thermo X Series II).

4.1 Composition and general properties of the thermal spring pool Bagnaccio TW

The values ​​of the temperature (Table A) are smaller in winter (23° C) and autumn (28.5° C), higher in spring (31.5° C) and summer (35° C). These variations are related to the seasonal rainfalls which reflect on the temperature reading. The pH is slightly acid and the chemical constituents further indicate that the thermal water is rich in sulphate and in earth alkaline elements with a clear prevalence of calcium and magnesium with respect to sodium and potassium. Minor elements are characterized by the high value of strontium followed, in descending order, by manganese, rubidium, cesium, arsenic, lithium, barium, aluminum, iron, vanadium, uranium (Table A). The analytical data were broadly consistent and stable during the sampling period. The values ​​of the isotopic ratio of strontium between 0.70822 and 0.70850 indicate that the thermal water was in contact with the evaporite rocks of the upper Triassic Calcare cavernoso and Anhydrites of Burano Formation in the lower part of Tuscan Nappe, recognized by the drilling Bagnaccio in the subsurface of geothermal area.

4.2 Composition and general properties of seawater (SW) from a representative Mediterranean area, Montalto di Castro

The seawater sample of Montalto di Castro has an elemental composition that is characteristic of the Italian seawaters of the Tyrrhenian area of the Mediterranean area. The pH is slightly basic, sodium and chloride ions show a clear prevalence. Data are in Table B.

**SI # 5: Mass-to-charge ratio** **data**.

Mass-to-charge ratio and the abundance of mass spectra peaks of compounds **1**-**27** are reported in Table C. Products were analysed after treatment with N,N-bis-trimethylsilyltrifluoroacetamide and pyridine. The degree of silylation for any product is reported in note.

**References**

41. Miller, S. L., Urey, H. C. Organic Compound Synthesis on the Primitive Earth.*Science* **130**, 245–251 (1959).

42. Adande, G. R., Woolf, N. J., Ziurys, L. M. Observations of interstellar formamide: availability of a prebiotic precursor in the galactic habitable zone. *Astrobiology* **13**, 439-453 (2013).

43. López-Sepulcre, A. *et al*. Shedding light on the formation of the pre-biotic molecule formamide with ASAI *Mon. Not. R. Astrom. Soc*. **449**, 2438-2458 (2015).

44. Carota, E., Botta, G., Rotelli, L., Di Mauro, E., Saladino, R. Current Advances in Prebiotic Chemistry Under Space Condition. *Curr. Org. Chem.* **19**, 1963–1979 (2015).

45. Henderson-Sellers, A. & Schwartz, A. W. Chemical evolution and ammonia in the early Earth's atmosphere. *Nature* **287**, 526–528 (1980).

46. Miller, S. L. A production of amino acids under possible primitive earth conditions. *Science* **117**, 528–529 (1953).

47. Niether, D., Afanasenkau, D., Dhont, J. K. G., Wiegand, S. Accumulation of formamide in hydrothermal pores to form prebiotic nucleobases *Proc. Natl. Acad. Sci. U.S.A.* **113**, 4272-4277 (2016).

48. Sleep, N. H., Zahnle, K., Neuhoff, P. S. Initiation of clement surface conditions on the earliest Earth. *Proc. Natl. Acad. Sci. U.S.A.* **98**, 3666–3672 (2001).

49. Niether, D., Afasenkau, D., Dhont, J.K.G., Wiegand, S. Accumulation of NH_2_COH in hydrothermal pores to form prebiotic nucleobases. *Proc. Natl. Acad. Sci. USA* **113**(16), 4272-7 (2016).

50. Saladino, R., Crestini, C., Pino, S., Costanzo, G., Di Mauro, E. Formamide and the origin of life. *Phys. Life Rev.* **9**, 84-104 (2012).

51. Huss, G. R., Rubin, A. E., Grossman, J. N. Thermal Metamorphism in Chondrites in *Meteorites and the Early Solar System II*, D.S. Lauretta, H.Y. McSween Jr., Eds. (Tucson: Univ. Arizona Press, 2006), 567-586.

52. Burton, A. S. *et al*. You have full text access to this contentAmino acid analyses of R and CK chondrites. *Meteorit. Planet. Sci.* **50**, 470-482 (2015).

53. Campbell, A. J., Humayun, M. Formation of metal in the CH chondrites ALH 85085 and PCA 91467. *Geochim. Cosmochim. Acta* **67**, 2481-2495 (2003).

54. Cloutis, E., Hudon, P., Hiroi, T., Gaffey, M., Mann P. Spectral reflectance properties of carbonaceous chondrites: 8. ‘‘Other’’ carbonaceous chondrites: CH, ungrouped, polymict, xenolithic inclusions, and R chondrites. *Icarus* **221**, 984-1001 (2012).

55. Grossman, J. N. The Meteoritical Bulletin, No. 76, 1994 January: The U.S. Antarctic Meteorite Collection. *Meteoritics* 29, 100-143 (1994).

56. Botta, O., Bada, J.L. Extraterrestrial organic compounds in meteorites. *Survey Geophysics* **23**, 411-467 (2002).

57. Pizzarello, S., Yarnes, C.T. Enantiomeric excesses of chiral amines in ammonia rich carbonaceous meteorites. *Hearth Planetary Science Lett*. **443**, 176-184 (2016).

58. Pizzarello, S, Huang, Y., Alexandre, M. R. Molecular asymmetry in extraterrestrial chemistry: Insights from a pristine meteorite *Proc. Natl. Acad. Sci. U.S.A.* **105**, 3700–3704 (2008).

59. Pizzarello, S., Holmes, W. Nitrogen-containing compounds in two CR2 meteorites: 15N composition, molecular distribution and precursor molecules. *Geochim. Cosmochim. Acta* **73**, 2150–2162, (2009).

60. Cronin, J. R., Pizzarello, S. Amino acids in meteorites. *Adv.Space Res*. **3**, 5-18 (1983).

61. Pearson, V. K., Sephton, M. A., Franchi, I. A., Gibson, J. M., Gilmour, I. Carbon and nitrogen in carbonaceous chondrites: Elemental abundances and stable isotopic compositions. *Meteorit. Planetary Science* **41**, 1899–1918 (2006).

62. Bose, M., Floss, C., Stadermann, F.J., Stroud, R.M., Speck, A.K. Circumstellar and interstellar material in the CO3 chondrite ALHA77307: An isotopic and elemental investigation. *Geochim. Cosmochim. Acta*  **93**, 77-101 (2012).

63. Saladino, R., Crestini, C., Cossetti, C., Di Mauro, E., Deamer, D. Catalytic effects of Murchison Material: Prebiotic Synthesis and Degradation of RNA Precursors. *Orig. Life Evol. Biosph.* **41**, 437-451 (2011).

**SI # 6: Selected chromatograms for the condensation reactions with NH_2_CHO/water systems in the presence of ALH84028, GRO95566 and LAR04318**.

Figure 2. GC-MS profile: ALH84028 and distilled water


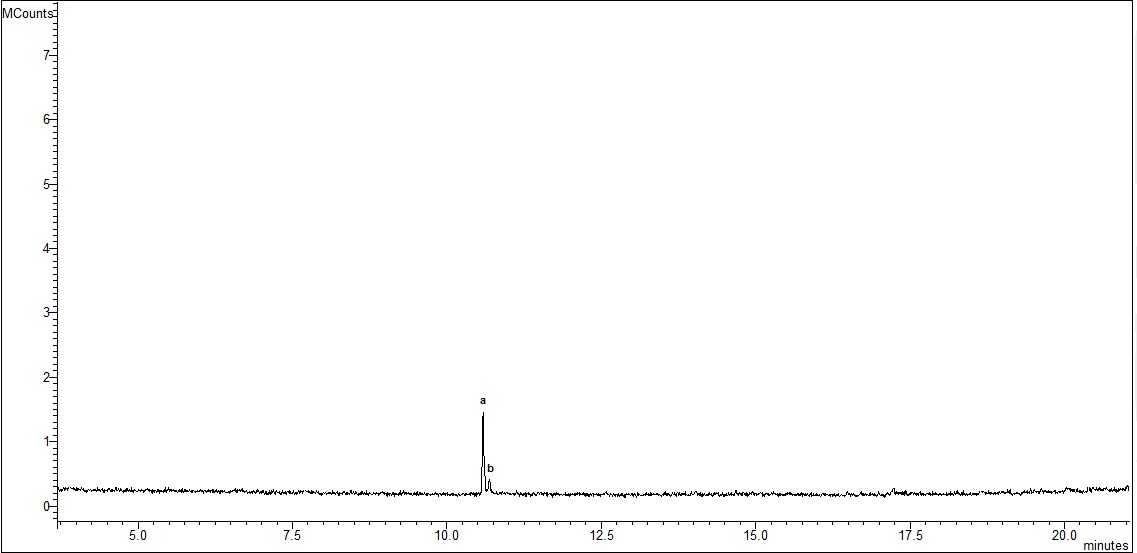


a and b: excess of N,N-bis-trimethylsilyltrifluoroacetamide

Figure 3. GC-MS profile: ALH84028, NH_2_CHO and distilled water


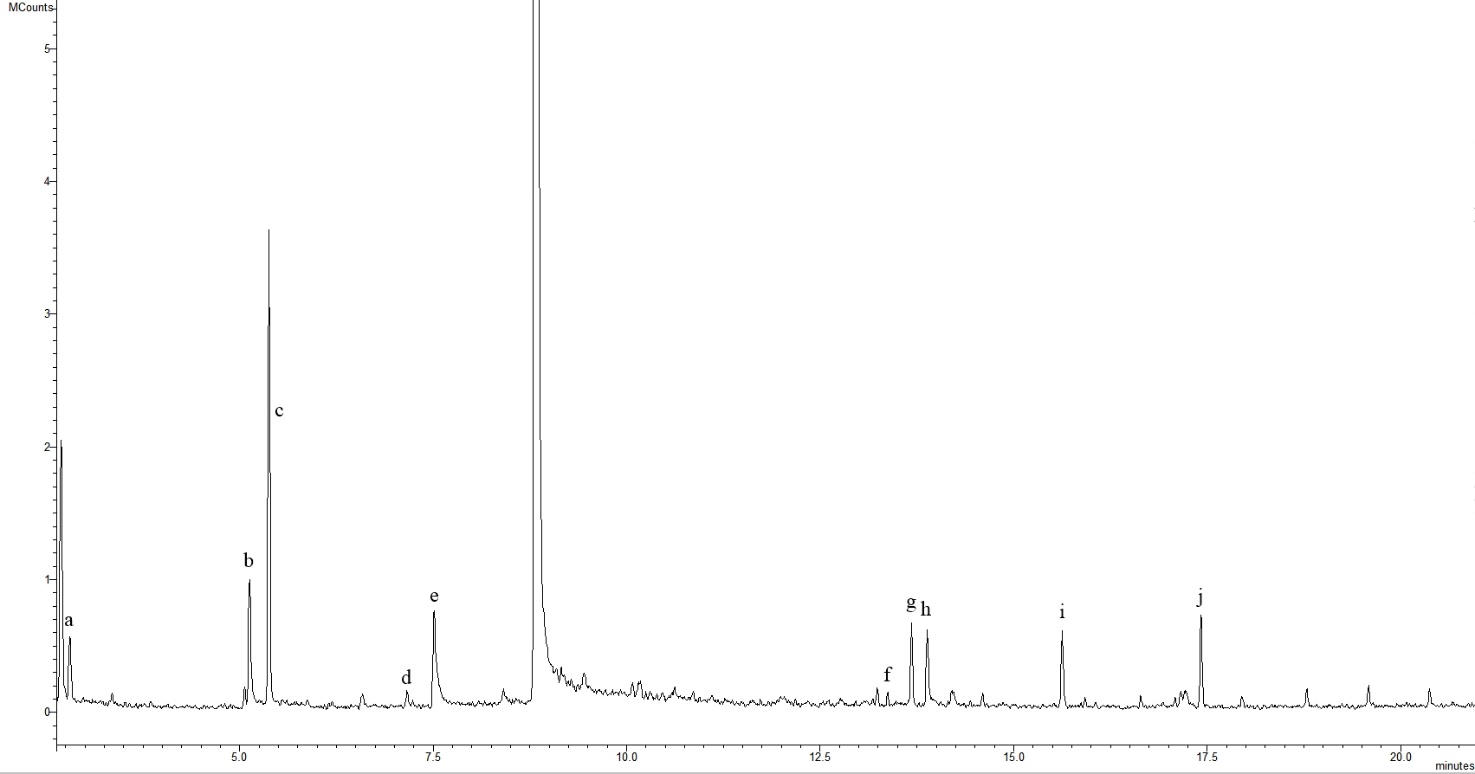


a, guanidine; b, oxalic acid; c, urea; d, parabanic acid; e, glycine; f, citric acid; g, fructose; h, glucose; i, palmitic acid; j, stearic acid.

Figure 4. GC-MS profile: ALH84028 and neat NH_2_CHO


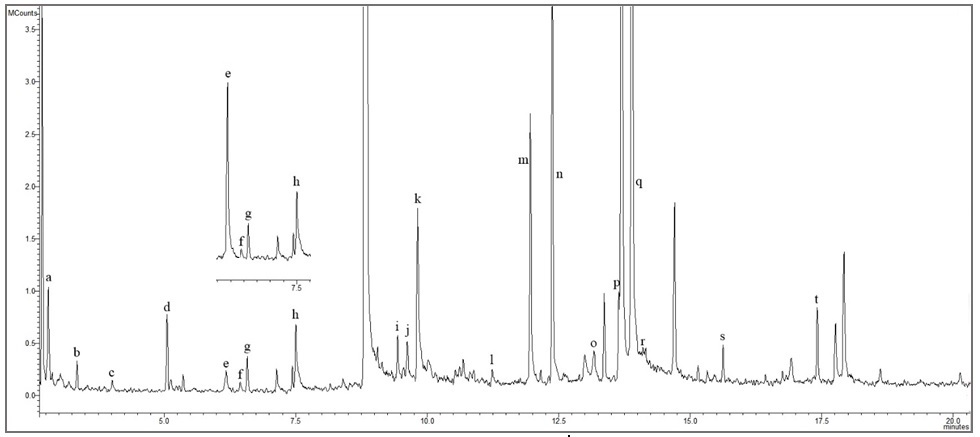


a, guanidine; b, succinic acid; c, oxalic acid; d, glycolic acid; e, urea; f, pyruvic acid; g, glycine; h, N-formylglycine; i, parabanic acid; j, uracil; k, purine; l, isocytosine; m, 2,4-diamnino-6-hydroxypyrimidine; n, 2,4-dihydroxypyrimidine-5-carboxylic acid; o, hypoxanthine; p, cytosine; q, adenine; r, citric acid; s, palmitic acid; t, stearic acid. The inset shows the magnification of a selected part of the chromatographic profile after addition of urea (10 μg) as an internal standard.

Figure 5. GC-MS profile: ALH84028 and TW


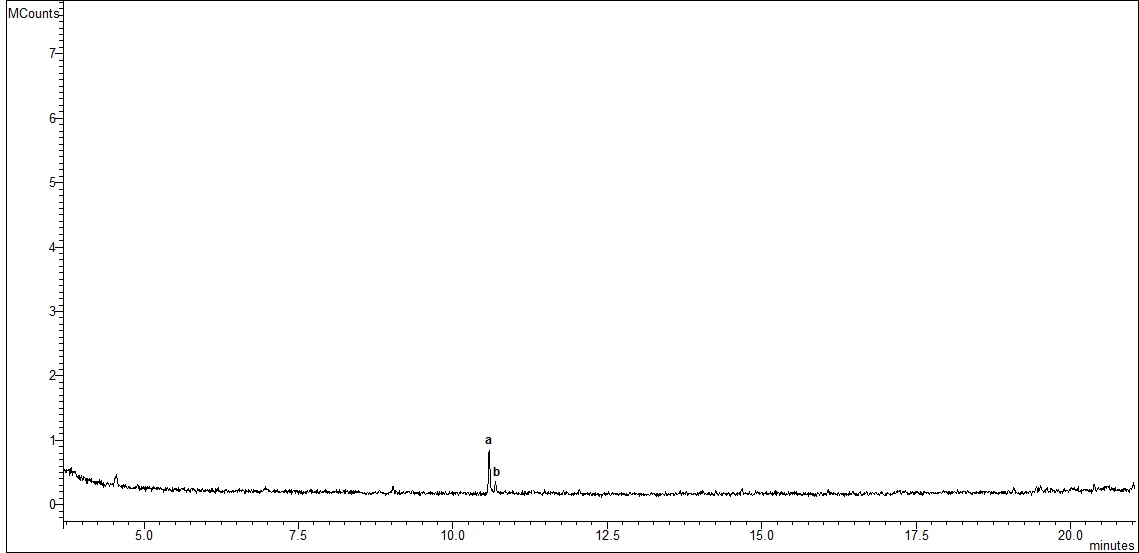


a and b: excess of N,N-bis-trimethylsilyltrifluoroacetamide


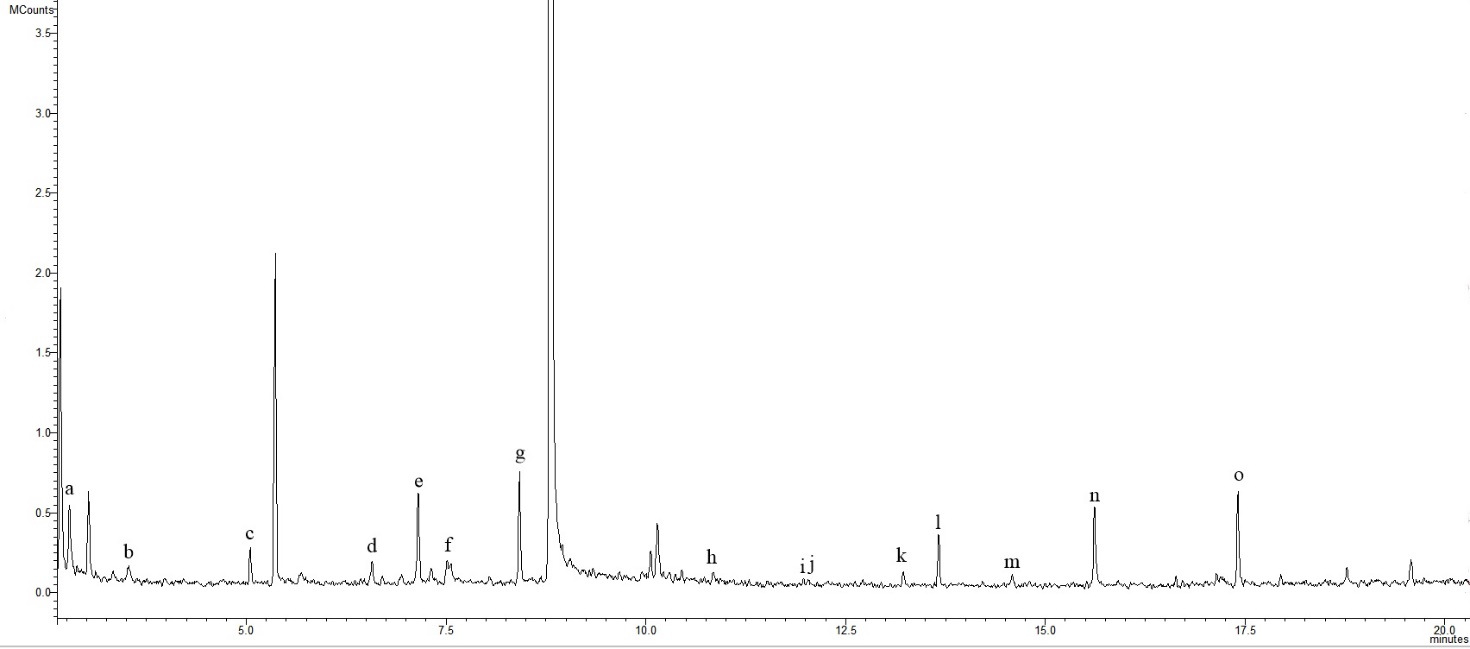
Figure 6. GC-MS profile: ALH84028, NH_2_CHO and TW

a, guanidine; b, oxalic acid; c, glycolic acid; d, pyruvic acid; e, glycine; f, *N*-formylglycine; g, uracil; h, 2,4-dihydroxypyrimidine-5-carboxylic acid; i, adenine; j, guanine; k, citric acid; l, fructose; m, probably sugar; n, palmitic acid; o, stearic acid.

Figure 7. GC-MS profile: ALH84028 and SW


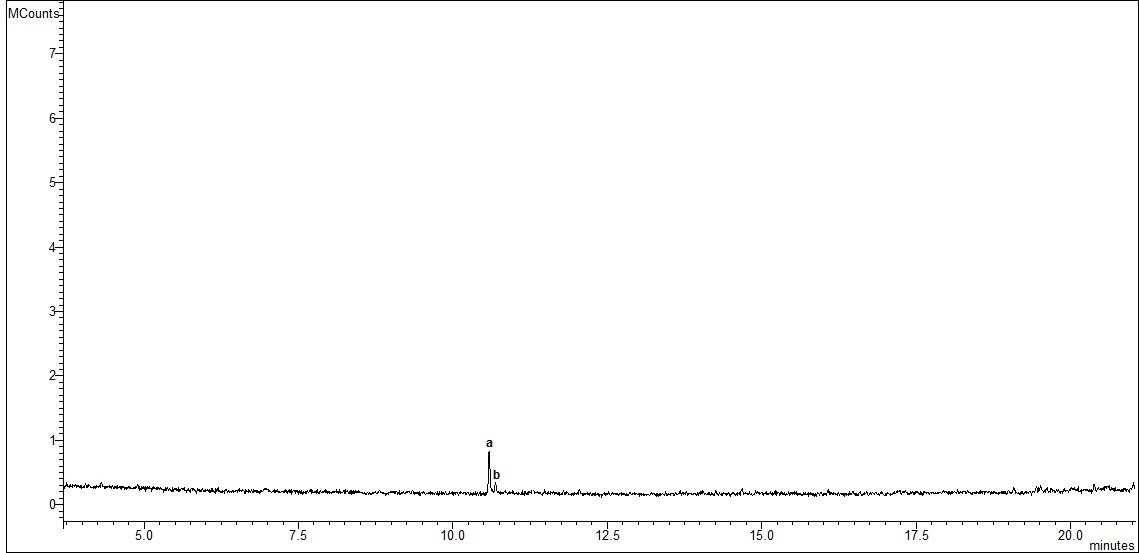


a and b: excess of N,N-bis-trimethylsilyltrifluoroacetamide

Figure 8. GC-MS profile: ALH84028, NH_2_CHO and SW


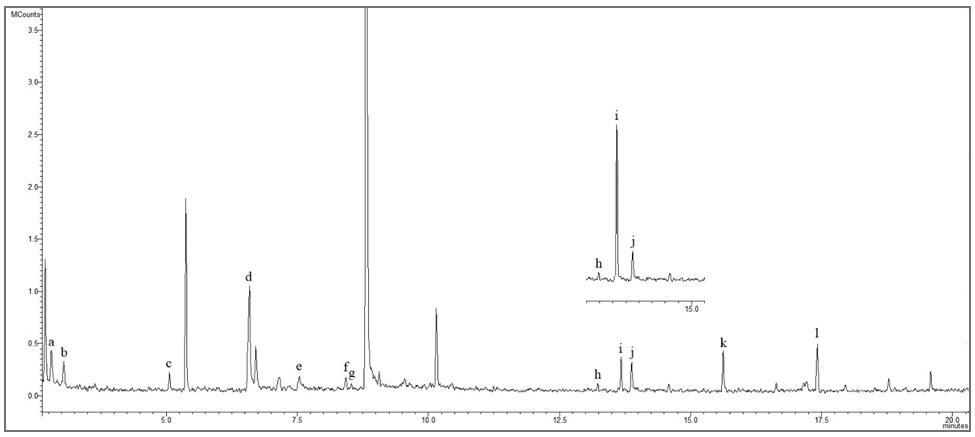


a, guanidine; b, oxalic acid; c, glycolic acid; d, glycine; e, *N*-formylglycine; f, parabanic acid; g, uracil; h, citric acid; i, adenine; j, guanine; k, palmitic acid;l, stearic acid. The inset shows the magnification of a selected part of the chromatographic profile after addition of adenine (10 μg) as an internal standard.


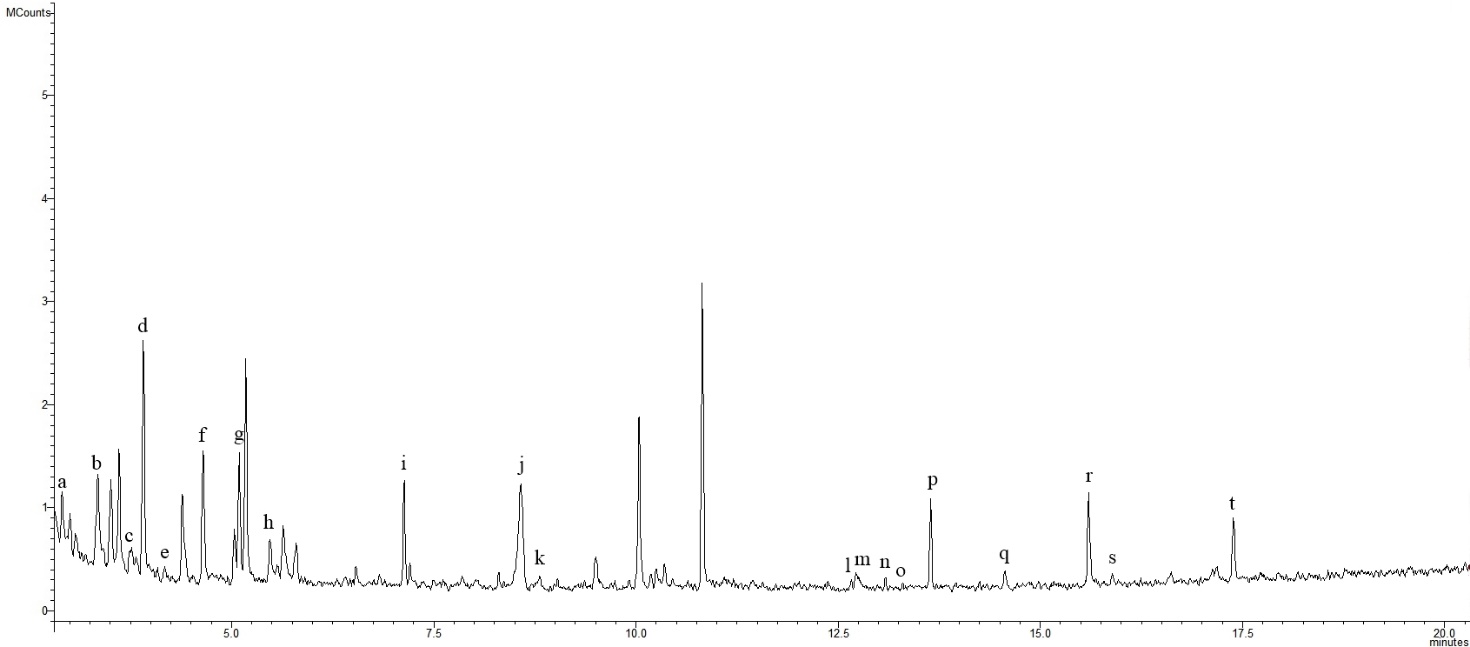


Figure 9. GC-MS profile: GRO95566 and neat NH_2_CHO

a, guanidine; b, succinic acid; c, lactic acid; d, pyruvic acid; e, oxaloacetic acid; f, 2,4-diamnino-6-hydroxypyrimidine; g, glycolic acid; h, 4(3H)-pyrimidinone; i, glycine; j, uracil; k, isocytosine; l, adenine; m, guanine; n, hypoxanthine; o, 2,4-dihydroxypyrimidine-5-carboxylic acid; p, probably sugar; q, probably sugar; r, palmitic acid; s, 2,6-diaminopurine; t, stearic acid.

Figure 10. GC-MS profile: GRO95566, NH_2_CHO and TW


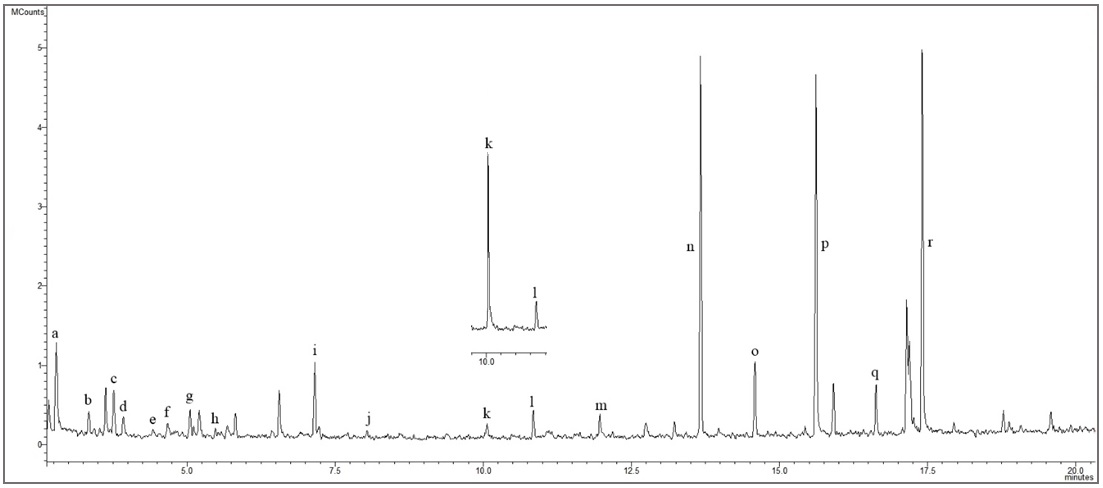


a, guanidine; b, succinic acid; c, lactic acid; d, pyruvic acid; e, oxaloacetic acid; f, 2,4-diamnino-6-hydroxypyrimidine; g, glycolic acid; h, 4(3H)-pyrimidinone; i, glycine; j, uracil; k, adenine; l, guanine; m, hypoxanthine; n, probably sugar; o, probably sugar; p, palmitic acid; q, 2,4-dihydroxypyrimidine-5-carboxylic acid; r, stearic acid. The inset shows the magnification of a selected part of the chromatographic profile after addition of adenine (10 μg) as an internal standard.


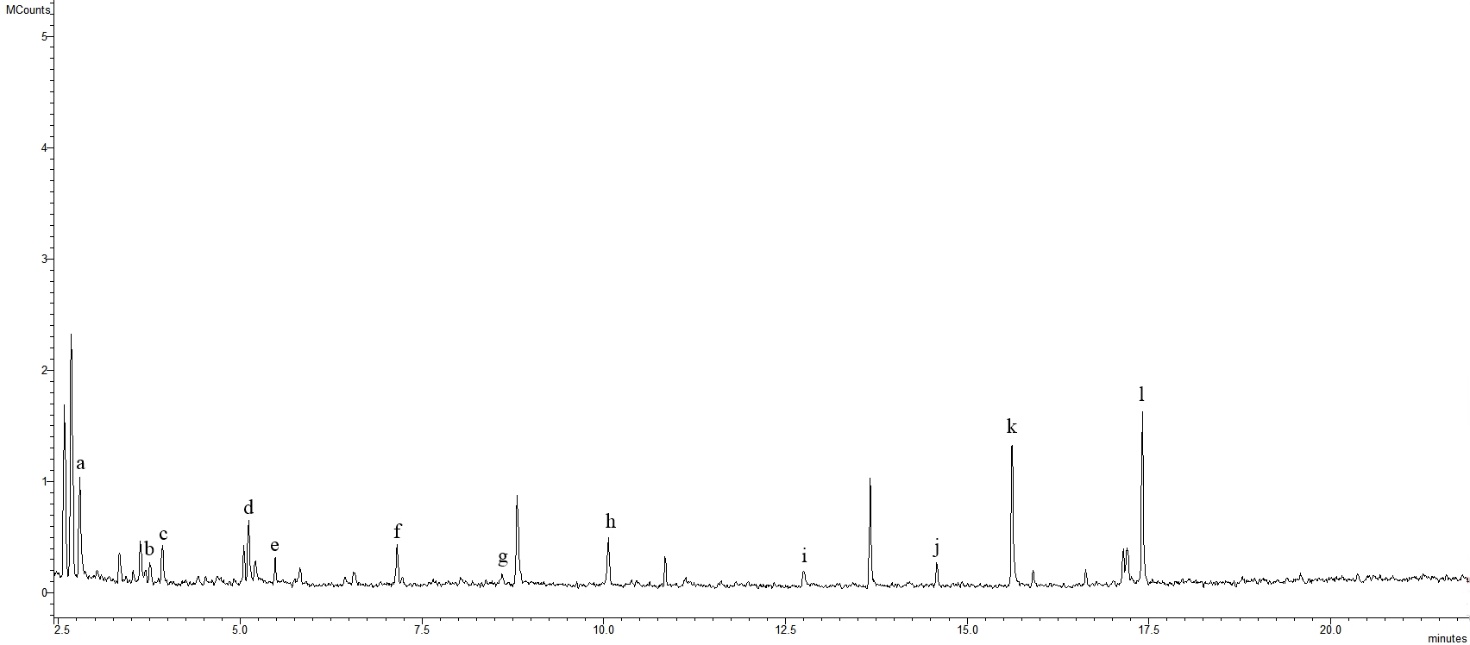
Figure 11. GC-MS profile: GRO95566, NH_2_CHO and SW

a, guanidine; b, lactic acid; c, pyruvic acid; d, glycolic acid; e, 4(3H)-pyrimidinone; f, glycine; g, uracil; h, *N*-formylglycine; i, guanine; j, probably sugar; k, palmitic acid; l, stearic acid.

Figure 12. GC-MS profile: LAR04318 and neat NH_2_CHO


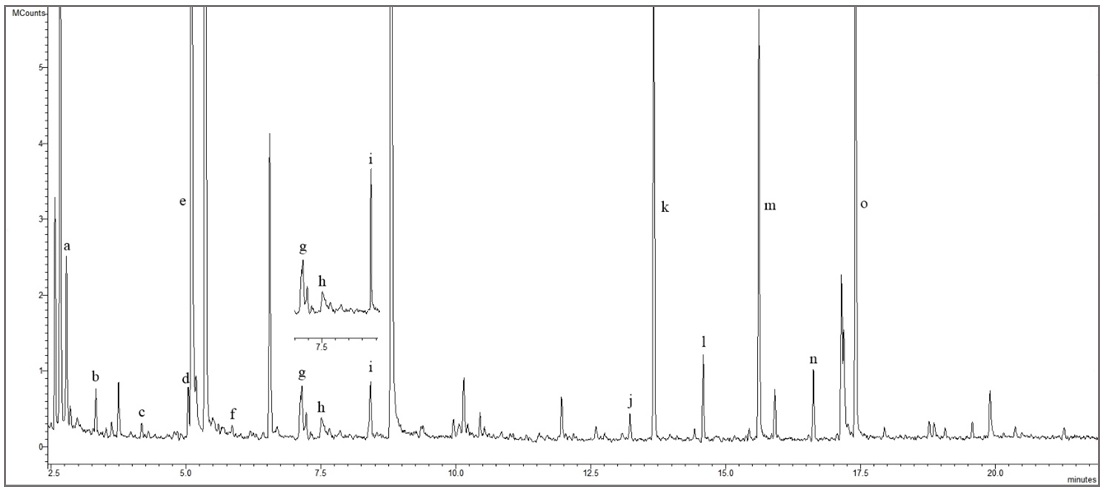


a, guanidine; b, succinic acid; c, 4(3H)-pyrimidinone; d, lactic acid; e, oxalic acid; f, urea; g, pyruvic acid; h, *N*-formylglycine; i, uracil; j, citric acid; k, fructose; l, probably sugar; m, palmitic acid; n, 2,4-dihydroxypyrimidine-5-carboxylic acid; o, stearic acid. At the top the magnification of a selected part of the chromatographic profile after addition of uracil (10 μg) as an internal standard.


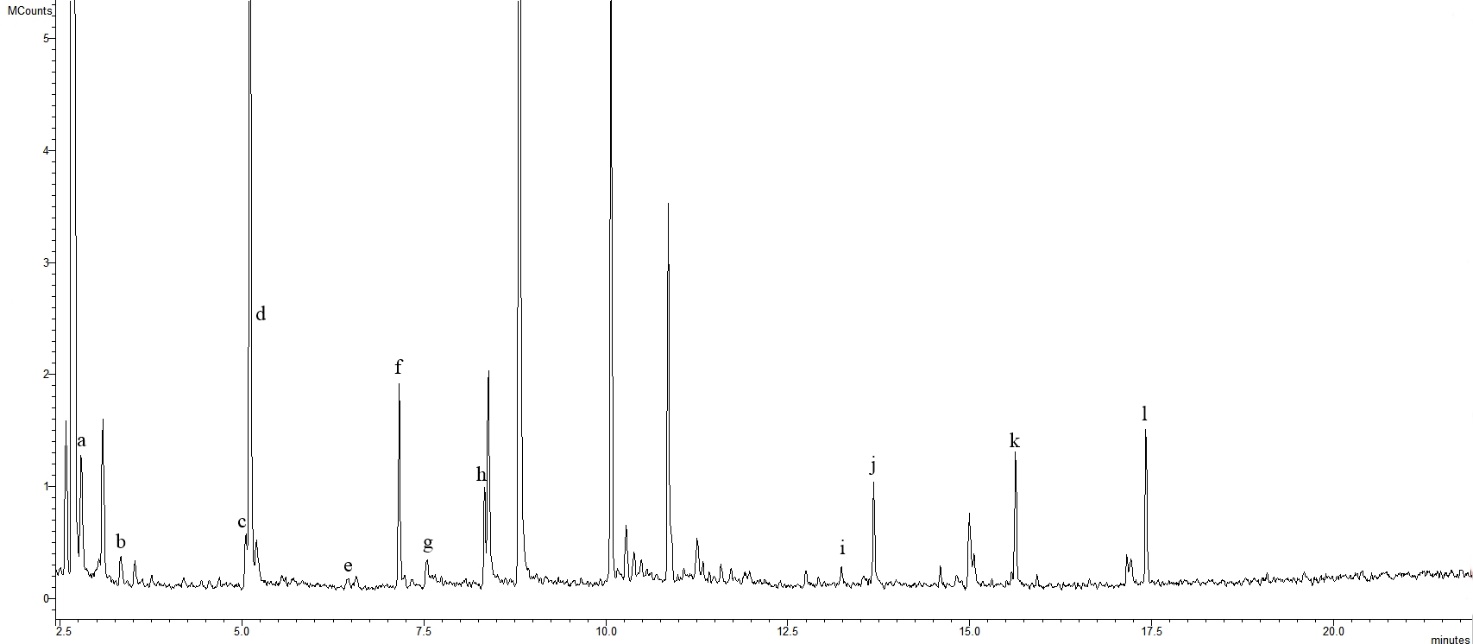
Figure 13. GC-MS profile: LAR04318, NH_2_CHO and TW

a, guanidine; b, succinic acid; c, lactic acid; d, oxalic acid; e, urea; f, pyruvic acid; g, glycine; h, *N*-formylglycine; i, citric acid; j, probably sugar; k, palmitic acid; l, stearic acid.

Figure 14. GC-MS profile: LAR04318, NH_2_CHO and SW


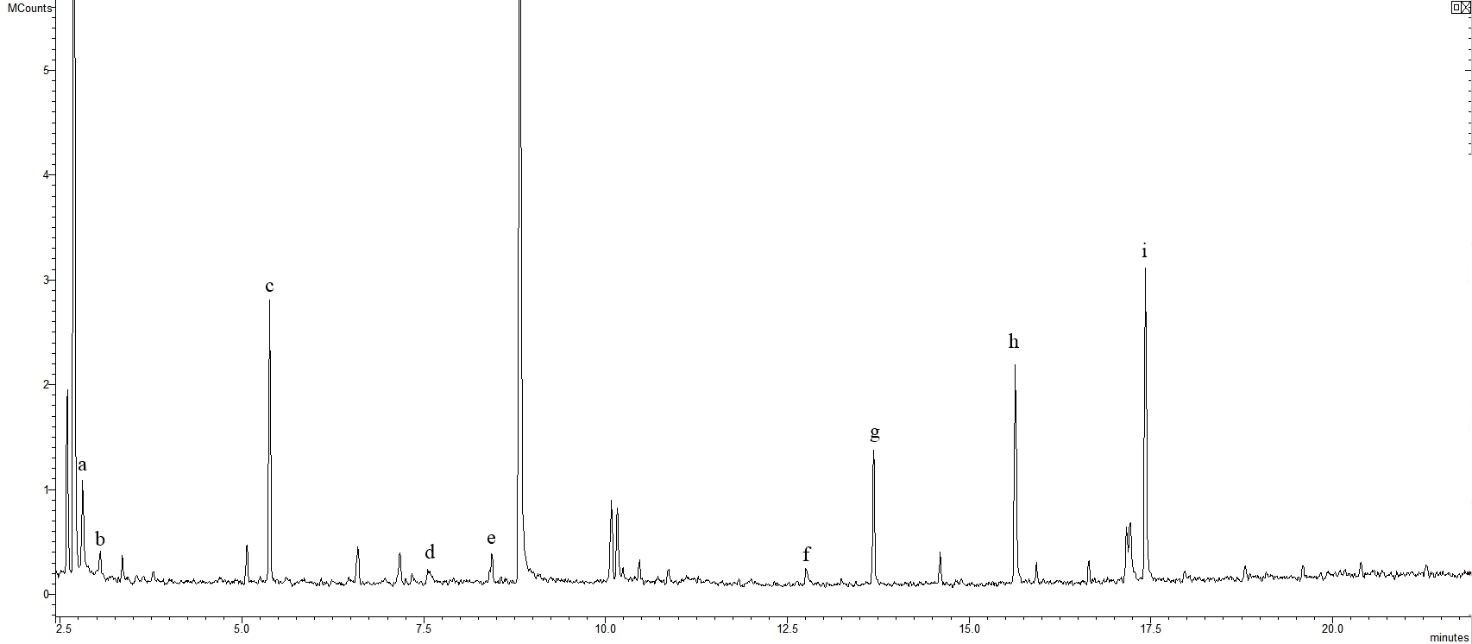


a, guanidine; b, succinic acid; c, oxalic acid; d, glycine; e, *N*-formylglycine; f, citric acid; g, probably sugar; h, palmitic acid; i, stearic acid.

**
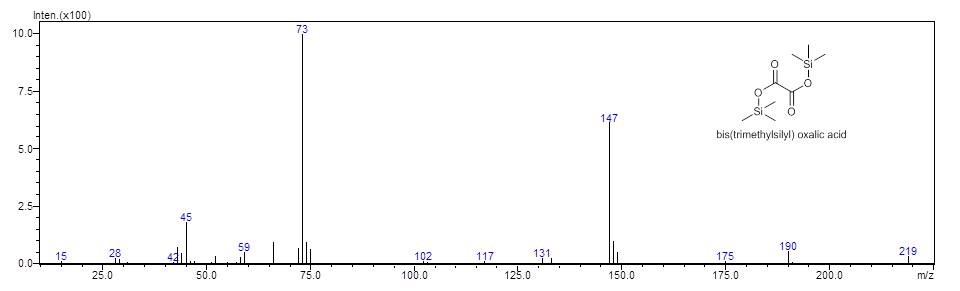
S.I. # 7: Mass-fragmentation spectra of compounds 1-23.** All products have been recognized with a similarity index (S.I.) greater than 98% compared to reference standards.

Mass-Fragmentation spectrum : Bis (trimethylsilyl) oxalic acid

**
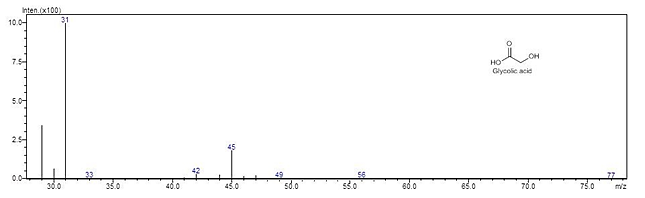
**

Mass-Fragmentation spectrum : Glycolc acid

Mass-Fragmentation spectrum : (Trimethylsilyl) piruvic acid

**
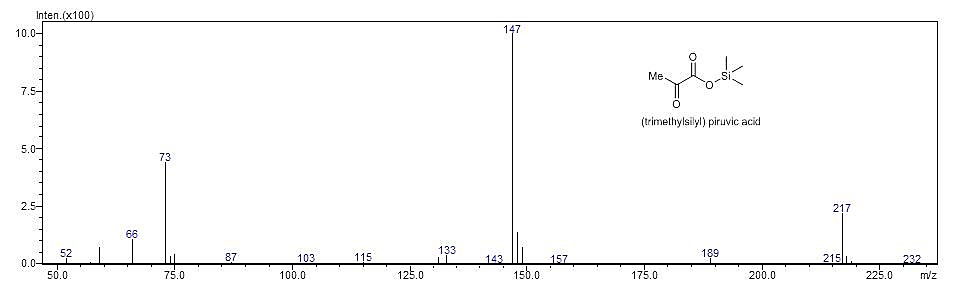
**

Mass-Fragmentation spectrum : Bis (trimethylsilyl) lactic acid

**
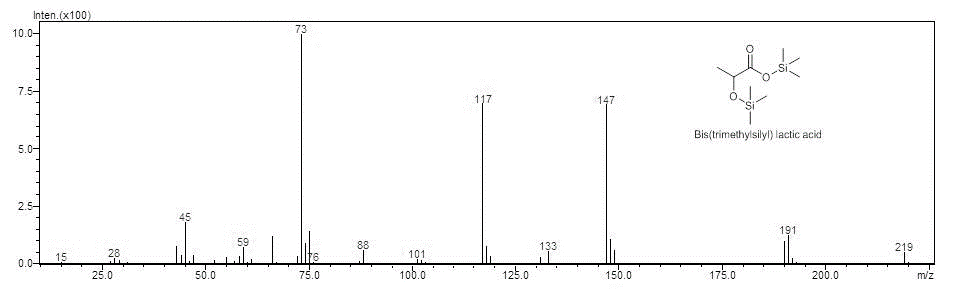
**

Mass-Fragmentation spectrum : Bis (trimethylsilyl) parabanic acid


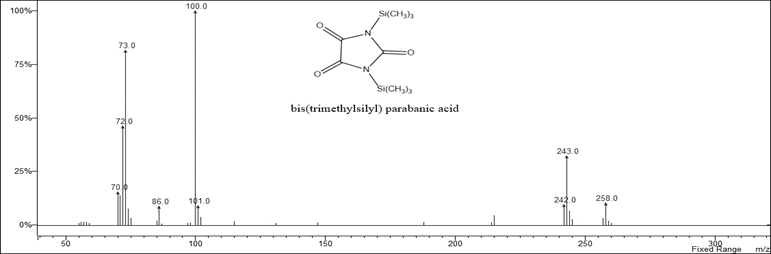


Mass-Fragmentation spectrum : Bis (trimethylsilyl) succinic acid

**
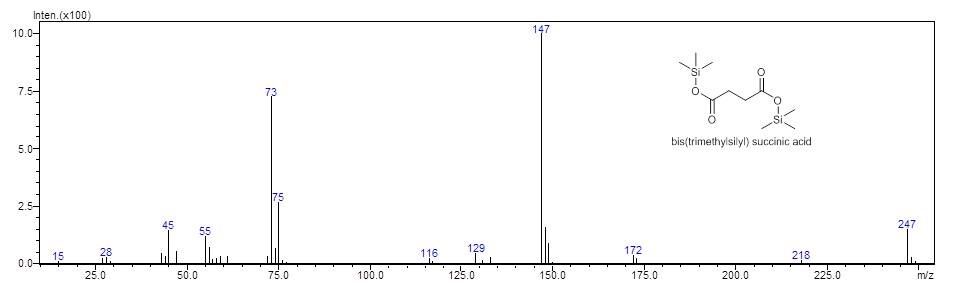
**

Mass-Fragmentation spectrum : Trimethylsilyl palmitic acid


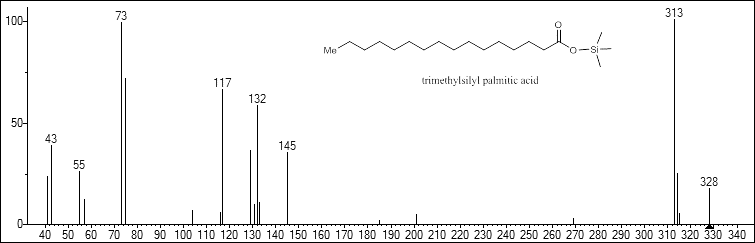


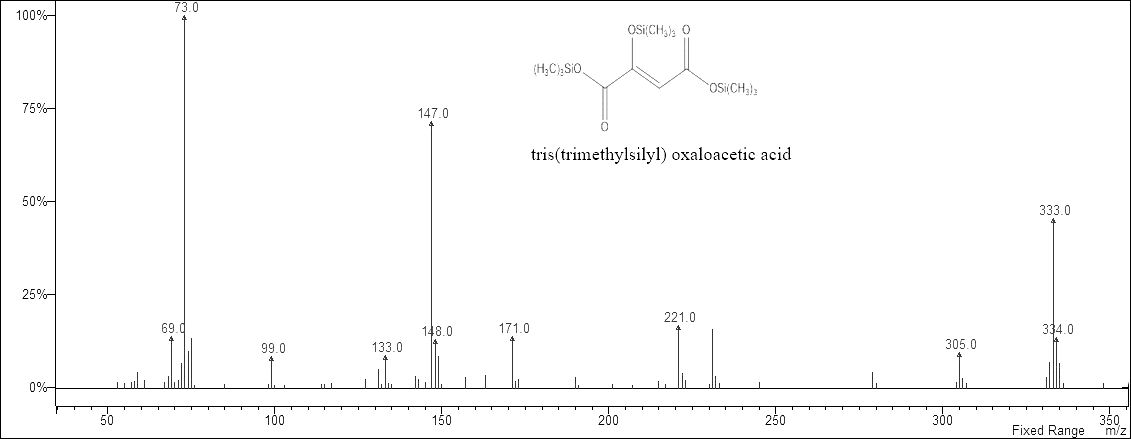


Mass-Fragmentation spectrum : Tris (trimethylsilyl) oxaloacetic acid

Mass-Fragmentation spectrum : Trimethylsilyl stearic acid


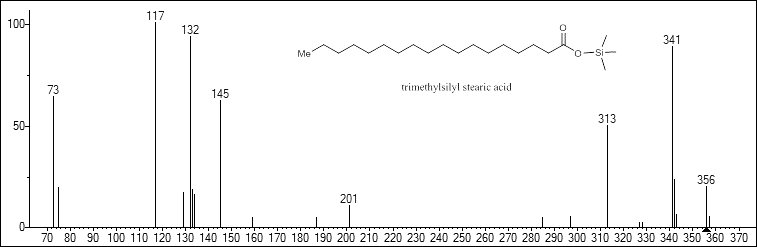


Mass-Fragmentation spectrum : Tetra (methylsilyl) citric acid


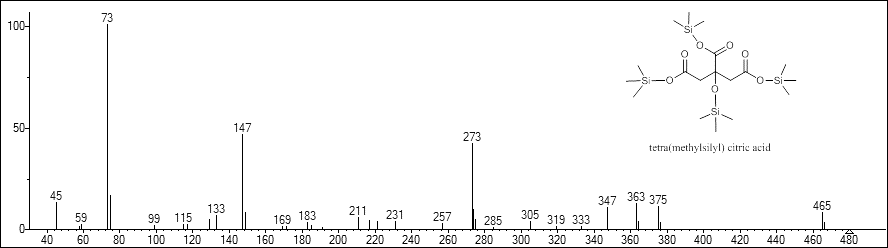


Mass-Fragmentation spectrum : Bis (trimethylsilyl) uracil

**
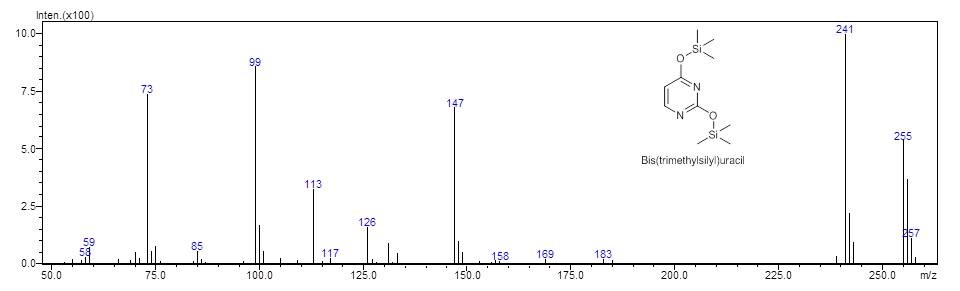
**

Mass-Fragmentation spectrum : N, 9–bis (trimethylsilyl) adenine

**
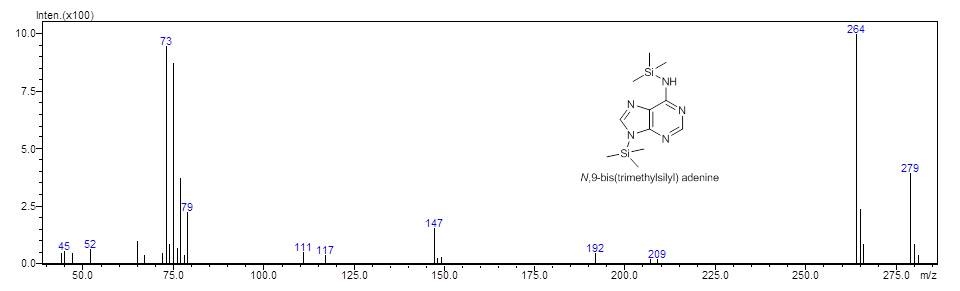
**

Mass-Fragmentation spectrum : Guanine

**
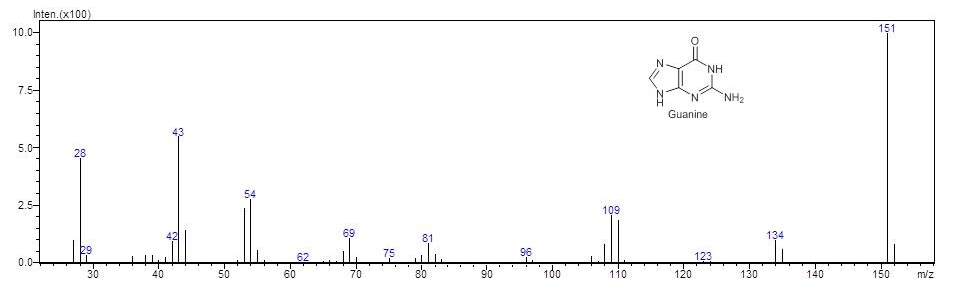
**

Mass-Fragmentation spectrum : 9-(trimethylsilyl)-6-(trimethylsilyl hypoxantine

**
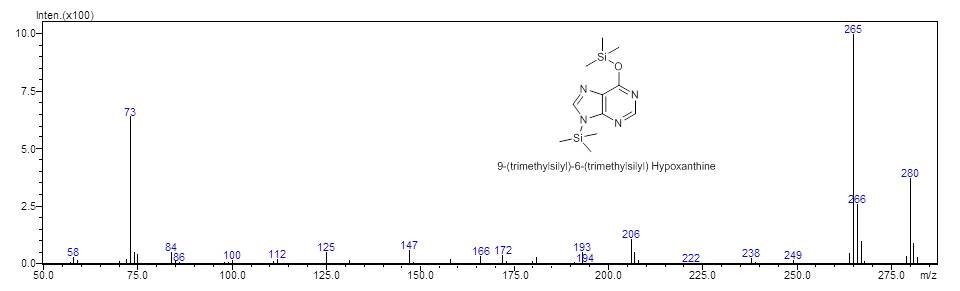
**

Mass-Fragmentation spectrum : Bis (trimethylsilyl) isocytosine

**
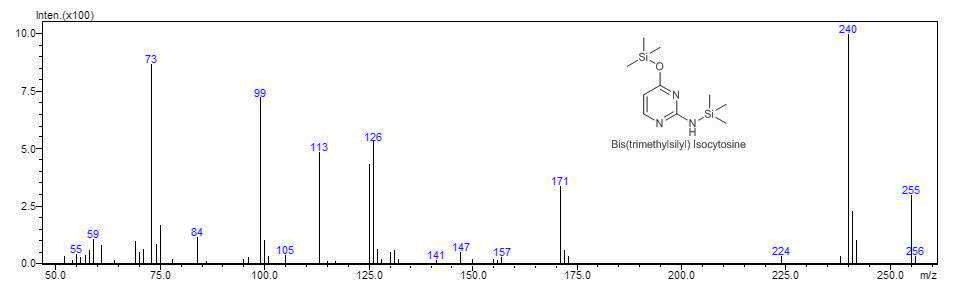
**

Mass-Fragmentation spectrum : Tris (trimethylsilyl) 2,4-dihydroxypyrimidine-5-carboxylic acid


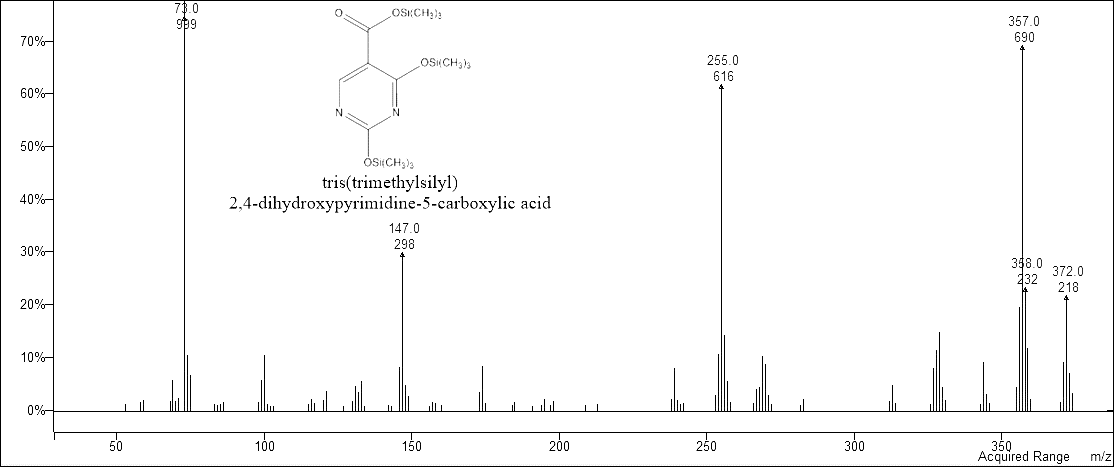


Mass-Fragmentation spectrum : Bis (trimethylsilyl) 2,4-diammino-6-hydroxypyrimidine


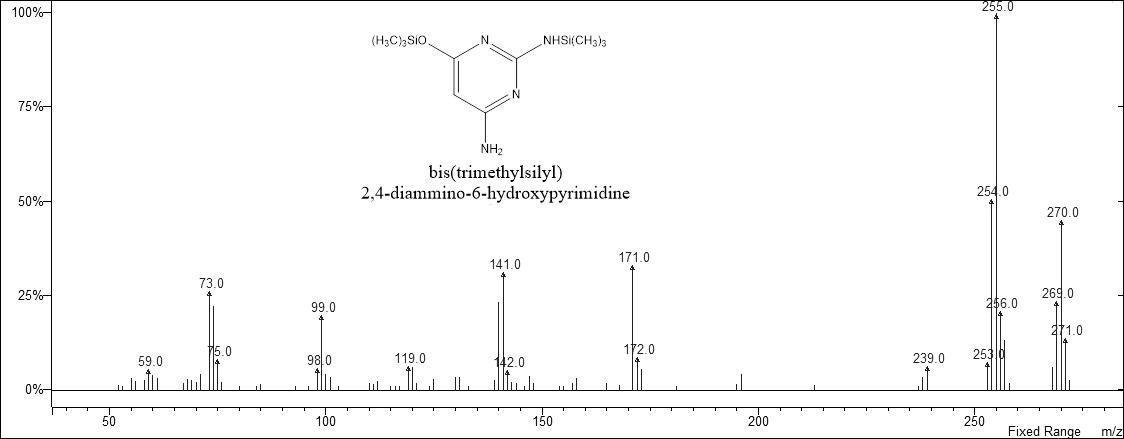


Mass-Fragmentation spectrum : Tris (trimethylsilyl) 2,6-diaminopurine


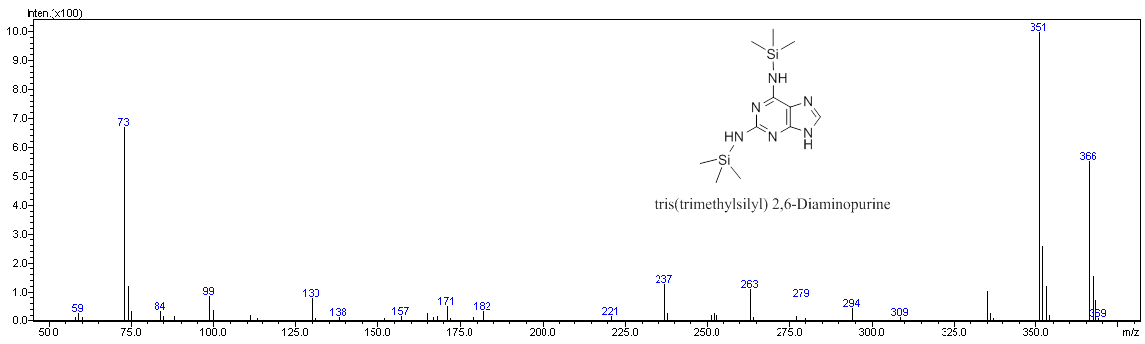


Mass-Fragmentation spectrum : trimethylsilyl-4(3H)-pyrimidinone


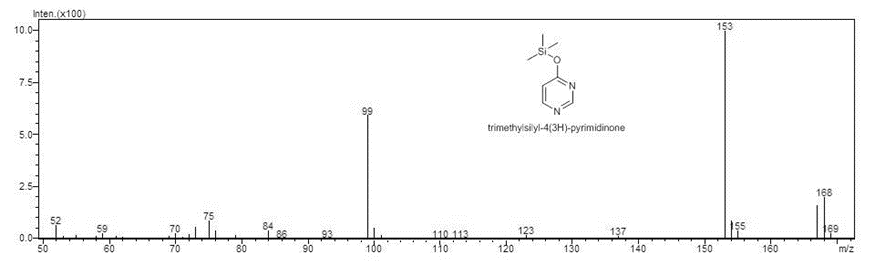


Mass-Fragmentation spectrum : 1,3-bis(trimethylsilyl)urea

**
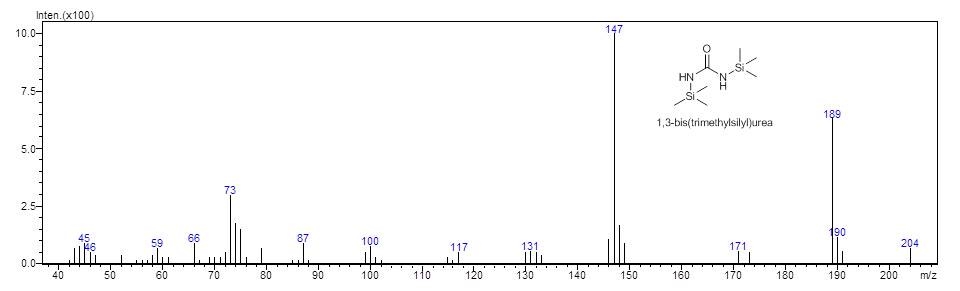
**

Mass-Fragmentation spectrum : 1,3-bis(trimethylsilyl)guanidine

**
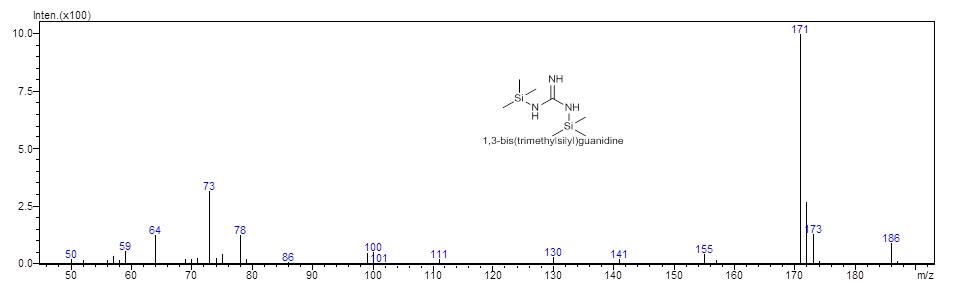
**

Mass-Fragmentation spectrum : trimethylsilyl glycine

**
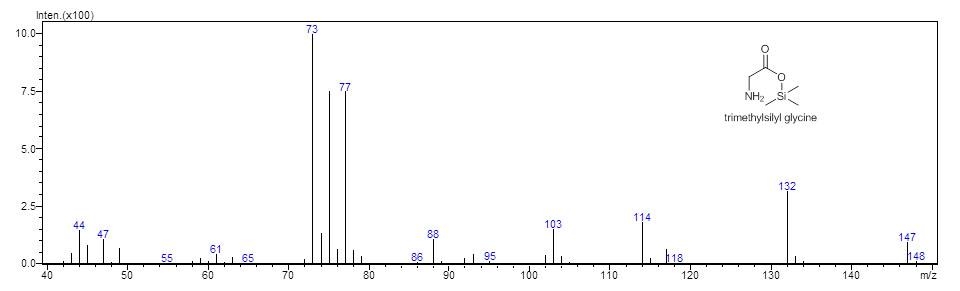
**

Mass-Fragmentation spectrum : triemthylsilyl N-formyl glycine

**
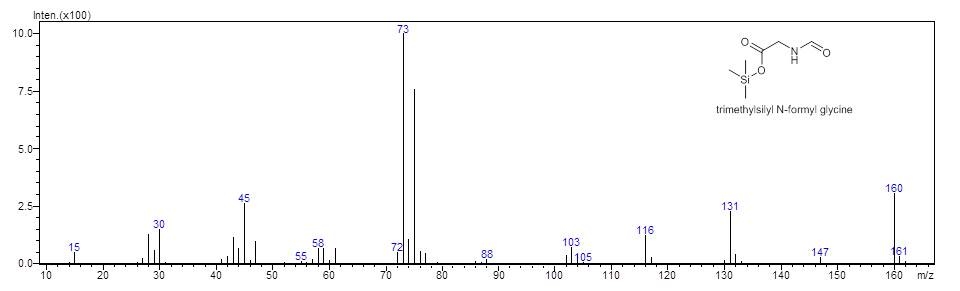
**

Table A. Results of the chemical-physic analyses of thermal water (TW) of the spring pool Bagnaccio (samples **I**-**IV**). The major elements are reported in mg/L, the trace and minor elements in mg/kg.

|  | **I** | **II** | **III** | **IV** | **Mean** |
| --- | --- | --- | --- | --- | --- |
| **T °C** | 23 | 31.5 | 35 | 28.5 | 29.5 |
| **pH** | 6.3 | 6.3 | 6.4 | 6.3 | 6.3 |
| **Ca** | 458.1 | 453.5 | 433.6 | 450.6 | 449.0 |
| **Mg** | 115.2 | 110.8 | 118.8 | 121.4 | 116.6 |
| **Na** | 13.5 | 12.1 | 14.3 | 12.3 | 13.1 |
| **K** | 28.7 | 26.7 | 25.8 | 28.8 | 27.5 |
| **Cl** | 15.4 | 16.8 | 18.5 | 20.5 | 17.8 |
| **SO_4_** | 1300 | 1350 | 1380 | 1420 | 1362.5 |
| **HCO_3_** | 335 | 348 | 360 | 320 | 340,8 |
| **F** | 1.9 | 2.1 | 2.2 | 2 | 2.1 |
| **NO_3_** | 9,9 | 10,4 | 9,6 | 10,3 | 10,1 |
| **TDS** | 2277 | 2320 | 2363 | 2386 | 2336.5 |
| **Li** | 13,13 | 11,1 | 12 | 11 | 11,8 |
| **V** | 0.2 | 1.2 | 1.7 | 2.2 | 1.3 |
| **Mn** | 29,6 | 26,2 | 24,4 | 25,1 | 26,3 |
| **Fe** | 0.3 | 0.1 | 0.4 | 0.8 | 0.4 |
| **As** | 15 | 25 | 22,2 | 25,1 | 21,8 |
| **Rb** | 24.5 | 33.4 | 31.8 | 33.1 | 30.7 |
| **Sr** | 806.8 | 950 | 988 | 970 | 928.7 |
| **Cs** | 17.6 | 15.1 | 12.4 | 11.2 | 14.1 |
| **Ba** | 6.9 | 8.3 | 9.3 | 9.8 | 8.6 |
| **U** | 0,1 | 0,1 | 0,2 | 0,4 | 0,2 |
| **Al** | 1.6 | 1.4 | 2.1 | 3.1 | 2.1 |

Table B. Results of the chemical-physic analyses of seawater (SW) from a representative Mediterranean area, Montalto di Castro (Viterbo, Italy). The major elements are reported in g/L.

|  | **I** | **II** | **Mean** |
| --- | --- | --- | --- |
| **T °C** | 25 | 25 | 25 |
| **pH** | 8.1 | 8.0 | 8,05 |
| **Ca** | 0,40 | 0,39 | 0,395 |
| **Mg** | 1,27 | 1,25 | 1,26 |
| **Na** | 10,56 | 10,52 | 10,53 |
| **K** | 0,38 | 0,38 | 0,38 |
| **Cl** | 18,97 | 18,95 | 18,96 |
| **SO_4_** | 2,65 | 2,64 | 2,645 |
| **HCO_3_** | 0,14 | 0,13 | 0,135 |
| **F** | 0,001 | 0,001 | 0,001 |
| **Br** | 0,07 | 0,07 | 0,07 |
| **H_2_B0_3_** | 0,03 | 0,03 | 0,03 |

Table C: Mass-to-charge ratio (m/z) value and the abundance of mass spectra peaks of compounds.^[a]^

| Products | *m/z* (%) |
| --- | --- |
| Glycolic acid (**1**) | 205 (8) [M-CH_3_], 190 (1) [M-(CH_3_)_2_], 148 (10) [M-Si(CH_3_)_3_], 147 (74) [M-HSi(CH_3_)_3_], 133 (9) [M-CH_3_-Si(CH_3_)_3_], 117 (4) [M-(CH_3_)_2_-HSi(CH_3_)_3_], 103 (5) [M-(CH_3_)_3_-Si(CH_3_)_3_] |
| Oxalic acid (**2**)^[c]^ | 219 (3) [M-CH_3_], 189 (5) [M-(CH_3_)_3_], 147 (78) [M-Si(CH_3_)_3_-CH_3_], 117 (1) [M-Si(CH_3_)_3_-3xCH_3_], 73 (100) |
| Piruvic acid (**3**)^[c]^ | 160 (10) [M], 145 (7) [M-CH_3_], 88 (14) [M-Si(CH_3_)_3_], 71 (12) [M-Si(CH_3_)_3_-OH], 43 (100) [M-HSi(CH_3_)_3_-CO_2_] |
| Lactic acid (**4**)^[c]^ | 219 (6) [M-CH_3_], 190 (14) [M-CO_2_] , 147 (71) [M-Si(CH_3_)_3_-CH_3_], 133 (7), 117 (76) [M-Si(CH_3_)_3_-(CH_3_)_3_] |
| Parabanic acid (**5**) | 258 (15) [M], 243 (35) [M-CH_3_], 215 (18) |
| Malic acid (**6**)^[c]^ | 278 (65) [M], 206 (100) [M- Si(CH_3_)_3_], 191 (23) [M-Si(CH_3_)_3_-CH_3_], 162 (35) [M- Si(CH_3_)_3_-CO_2_] |
| Succinic acid (**7**)^[c]^ | 247 (16) [M-CH_3_], 173 (5) [M-HOSi(CH_3_)_3_], 147 (100), 73 (80) |
| Oxaloacetic acid (**8**)^[d]^ | 333 (10) [M-CH_3_], 231 (11) [M-HOSi(CH_3_)_3_-CO], 158 (7), 147 (80), 73 (100) |
| Fumaric acid (**9**) ^[c]^ | 245 (18), 147 (100), 73 (80), 45 (22) |
| Ketoglutaric acid (**10**) ^[c]^ | 347 (6) [M-CH_3_], 272 (4) [M-HOSi(CH_3_)_3_], 245 (7) [M-OSi(CH_3_)_3_-CO], 147 (50), 73 (100) |
| Citric acid (**11**)^[d]^ | 465 (8) [M-CH_3_], 375 (10) [M-7(CH_3_)_3_], 363 (11) [M-Si(CH_3_)_3_-3CH_3_], 273 (45) [M-2HSi(CH_3_)_3_-(CH_3_)_3_- CH_3_] |
| Palmitic acid (**12**)^[b]^ | 328 (20) [M], 313 (100), [M-CH_3_], |
| Stearic acid (**13**) | 356 (20) [M], 341 (90) [M-CH_3_], 284 (5)[M-Si(CH_3_)_3_], |
| Uracil (**14**)^[c]^ | 256 (35) [M], 241 (100) [M-CH_3_], 225 (15) [M-CH_3_-CH_4_], 182 (7) [M-Si(CH_3_)_3_-H_2_], 142 (70), 113 (55) |
| Adenine (**15**)^[c]^ | 279 (27) [M], 264 (100) [M-CH_3_], 249 (1) [M-(CH_3_)_2_], 192 (17) |
| Guanine (**16**) | 151 (100) [M], 134 (14) [M-NH_3_], 109 (28) |
| Hypoxanthine (**17**)^[c]^ | 280 (49) [M], 265 (100) [M-CH_3_], 193 (8), 182 (80) |
| Isocytosine (**18**)^[c]^ | 255 (49) [M], 254 (100) [M-H], 240 (72) [M-CH_3_], 182 (5) [M-HSi(CH_3_)_3_] |
| 2,6-Diaminopurine (**19**)^[d]^ | 279 (20) [M], 264 (100) [M-CH_3_], 207 (18) [M-Si(CH_3_)_3_] |
| 4(3H)pyrimidinone (**20**)^[b]^ | 168 (25) [M], 153 (100) [M-CH_3_], 123 (5) [M-(CH_3_)_3_], 99 (100) |
| Uracil 5-COOH (**21**)^[d]^ | 372 (20) [M], 357 (68) [M-CH_3_], 255 (60) [M-HSi(CH_3_)_3_-3CH_3_] |
| 2,4-diNH_2_-6-OHpyr (**22**)^[c]^ | 270 (40) [M], 255 (100), [M-CH_3_], 239 (5) [M-2(CH_3_)], 171 (30) [M-Si(CH_3_)_3_-2CH_3_] |
| Glycine (**23**)^[b]^ | 147 (11) [M], 132 (28) [M-CH_3_], 88 (9),73 (100) |
| *N*-Formylglycine (**24**)^[b]^ | 160 (38) [M-CH_3_], 147 (5) [M-CO], 131 (22) [M-CONH_2_], 102 (11) [M-Si(CH_3_)_3_], 73 (100) |
| Alanine (**25**) ^[c]^ | 218 (4) [M- CH_3_], 190 (6) [M-SiCH_3_], 147 (13), 116 (100) [M-OSi(CH_3_)_3_-CO], 73 (60) |
| Urea (**26**)^[c]^ | 204 (7) [M], 189 (73) [M-CH_3_], 147 (100), 73 (35) |
| Guanidine (**27**)^[c]^ | 188 (11) [M-CH_3_], 173 (10) [M-2xCH_3_], 171 (100), 73 (33) |

^[a]^Mass spectroscopy was performed by using a 450GC-320MS Varian. Samples were analyzed after treatment with *N,N*-bis-trimethylsilyltrifluoroacetamide and pyridine. The peak abundance is reported in parenthesis. ^[b]^ Product analyzed as the monosilyl derivative; ^[c]^ Product analyzed as the bis-silyl derivative; ^[d]^ Product analyzed as the tris-silyl derivative.

**S.I. #8: Reaction pathways leading to nucleobases, carboxylic acids and amino acids from NH_2_COH**.

**1** Carboximide alcohol, **2** dihidroglycine, **3** Glycine, **4** 2-imidoacetonitrile, **6** glyoxylic acid, **7** oxalic acid, **8** glycolic acid, **9** DAMN (diaminomaleonitrile), **11** pyruvate, **13** DAFN (diaminofumaronitrile), **15** cytosine, **16** uracil, **17** thyamine, **19** AICN (amino imidazole carbonitrile), **20** AICA (amino imidazole carboxy amide), **22** guanine, **23** f-AICA (formyl amino imidazole carboxamide), **24** hypoxantine, **26** adenine.

Pathway A: Eschenmoser, A. On a Hypothetical Generational Relationship between HCN and Constituents of the Reductive Citric Acid Cycle. *Chem. Biodivers*. **4**, 554–573 (2007).

Pathway B: Saitta, A. M. & Saija, F. Miller experiments in atomistic computer simulations. *Proc. Natl Acad. Sci. USA* **111**, 13768-13773 (2014).

Pathway C1: Hudson, J. S. et al A Unified Mechanism for Abiotic Adenine and Purine Synthesis in Formamide. *Angewandte Chem*. **51**, 5134-5137 (2012).

Pathway C 2: Ferus, M. *et al*. High Energy chemistry of formamide: a simpler way for nucleobase formation. *Proc. Natl. Acad. Sci. USA* **112**, 657-662 (2015).

Pathway C 3: Saladino, R. et al. One-pot TiO_2_ catalyzed synthesis of nucleic bases and acyclonucleosides from formamide: Implications for the origin of life. *ChemBiochem* **4**, 514-521 (2003).
